# Identification and functional validation of super-enhancers in *Arabidopsis thaliana*

**DOI:** 10.1073/pnas.2215328119

**Published:** 2022-11-21

**Authors:** Hainan Zhao, Mingyu Yang, Jade Bishop, Yuhan Teng, Yingxue Cao, Brandon D. Beall, Shuanglin Li, Tongxin Liu, Qingxi Fang, Chao Fang, Haoyang Xin, Hans-Wilhelm Nützmann, Anne Osbourn, Fanli Meng, Jiming Jiang

**Affiliations:** ^a^Department of Plant Biology, Michigan State University, East Lansing, MI 48824; ^b^Key Laboratory of Soybean Biology in Chinese Ministry of Education, Northeast Agricultural University, Harbin 150030, China; ^c^The Milner Centre for Evolution, Department of Biology and Biochemistry, University of Bath, Bath BA2 7AY, United Kingdom; ^d^Department of Biochemistry and Metabolism, John Innes Centre, Norwich Research Park Colney Lane, Norwich NR4 7UH, United Kingdom; ^e^Northeast Institute of Geography and Agroecology, Key Laboratory of Soybean Molecular Design Breeding, Chinese Academy of Sciences, Harbin 150081, China; ^f^Department of Horticulture, Michigan State University, East Lansing, MI 48824; ^g^Michigan State University AgBioResearch, East Lansing, MI 48824

**Keywords:** enhancer, super-enhancer, transcriptional regulation, CRISPR/Cas, biosynthetic gene cluster

## Abstract

Super-enhancers (SEs) are exceptionally large enhancers and are recognized to play prominent roles in cell identity in mammalian species. We surveyed the genomic regions containing large clusters of accessible chromatin regions (ACRs) marked by deoxyribonuclease (DNase) I hypersensitivity in *Arabidopsis thaliana*. We identified a set of 749 putative SEs, which have a minimum length of 1.5 kilobases and represent the top 2.5% of the largest ACR clusters. We demonstrate that the genomic regions associating with these SEs were more sensitive to DNase I than other nonpromoter ACRs. The SEs were preferentially associated with topologically associating domains. Furthermore, the SEs and their predicted cognate genes were frequently associated with organ development and tissue identity in *A. thaliana*. Therefore, the *A. thaliana* SEs and their cognate genes mirror the functional characteristics of those reported in mammalian species*.* We developed CRISPR/Cas-mediated deletion lines of a 3,578-bp SE associated with the thalianol biosynthetic gene cluster (BGC). Small deletions (131–157 bp) within the SE resulted in distinct phenotypic changes and transcriptional repression of all five thalianol genes. In addition, T-DNA insertions in the SE region resulted in transcriptional alteration of all five thalianol genes. Thus, this SE appears to play a central role in coordinating the operon-like expression pattern of the thalianol BGC.

## Significance

Super-enhancers (SEs) are exceptionally large enhancers and play prominent roles in cell type identity and function in mammalian species. We identified a set of 749 putative SEs in the model plant *Arabidopsis thaliana*. We demonstrate that the SEs share the functional characteristics of mammalian SEs*.*We developed several small deletions within an SE located in the middle of the thalianol biosynthetic gene cluster. The deletion lines show distinct phenotypic changes and transcriptional repression of all five thalianol cluster genes. Our results suggest that SEs play important roles in regulating genes associated with development and tissue identity and in coordinating the coexpression of gene clusters in *A. thaliana*.

Enhancers are *cis*-regulatory elements (CREs) that can increase the transcription of their cognate genes. Enhancers serve as the binding docks for transcription factors (TFs). These enhancer-bound TFs then recruit transcriptional cofactors and other regulatory proteins to activate and boost the transcription of their target genes (1). Since enhancers function independently of distance and location relative to their cognate promoters, they have been difficult to identify due to their unpredictable genomic positions. However, enhancers can now be effectively predicted using genomic and/or epigenomic datasets, based on criteria such as genomic signatures associated with open chromatin (or chromatin accessibility) (2–5), specific histone modification marks (6), and binding sites of transcriptional coactivators (1, 7). Genome-wide enhancer prediction and identification have previously been reported in several model animal and plant species (3, 4, 6, 8–13). We have previously demonstrated that candidate enhancers predicted based solely on open chromatin signatures in *Arabidopsis thaliana* and maize show 70–80% validation rates using reporter gene assays (4, 14, 15).

Super-enhancers (SEs) were first recognized in mammalian species as exceptionally large enhancers, consisting of a cluster of regular enhancers, or “constituent enhancers”, in close genomic proximity (3, 16, 17). In one pioneering paper, only 308 of the ~8,000 enhancers (~4%) identified in a human multiple myeloma cell line were defined as SEs (16). The median size of a typical enhancer in this study was found to be 1.3 kb. In contrast, SEs had a median size of 19.4 kb (16). Similarly, a total of 8,794 enhancers were identified in murine embryonic stem cells (ESCs), of which only 231 were identified as SEs (17). Here, the average sizes of the enhancers and SEs were 703 bp and 8.7 kb, respectively (17). The extraordinary size of SEs has been confirmed in many other mammalian cell lines (18). The genomic regions harboring mammalian SEs are associated with high levels of transcriptional coactivators, such as Mediator, as well as with the enhancer-associated chromatin mark H3K27ac (16, 17). SEs have been recognized to play prominent roles in cell identity in mammalian species. A substantial fraction of SE-cognate genes are cell type specific, and these genes, in a given cell type, are highly enriched for the biological processes that define the identity of that cell type (3, 19). Interestingly, disease-associated variation is especially enriched in SEs identified in disease-relevant cell types (3, 19). Therefore SEs play key roles in human cell identity in both health and in disease (3).

We were intrigued by the possibility of SE-mediated transcriptional regulation in plants. We surveyed the genomic regions containing large clusters of accessible chromatin regions (ACRs) marked by DNase I hypersensitive sites (DHSs) in *A. thaliana*. We identified a set of 749 putative SEs that represent the top 2.5% of the largest ACR clusters without overlapping with predicted promoters. These putative SEs and their predicted cognate genes mirrored the functional characteristics of SEs and cognate genes reported in mammalian species. We provide several lines of evidence to indicate that these SEs and their cognate genes likely play a major role in organ development and tissue identity in *A. thaliana*. We developed CRISPR/Cas-mediated deletion lines of a SE associated with the thalianol biosynthetic gene cluster (BGC) (20, 21). Remarkably, small deletions (131–157 bp) within the SE resulted in both phenotypic changes and transcriptional repression of all five thalianol cluster genes, providing evidence for a role for this SE in regulating the operon-like expression pattern of the five thalianol cluster genes.

## Results

### Putative SEs in *A. thaliana*.

**Fig. 1.**
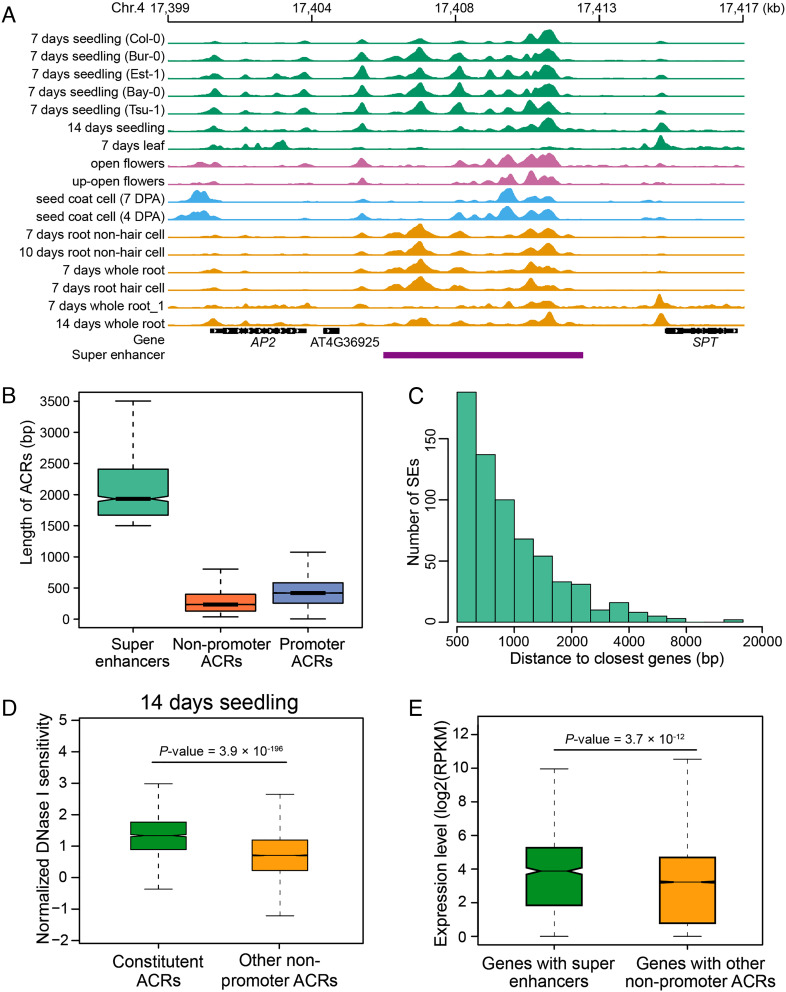
Genomic characteristics associated with SEs in *A. thaliana*. (*A*) A representative SE that spans 6.8 kb and is located between two well-studied TFs (AP2 and SPT) related to flower development. Each DNase-seq peak represents an independent ACR. A total of 13 independent ACRs were identified from 17 different DNase-seq datasets derived from leaf or seedling (green), flowers (purple), seed coat (blue), and root (brown) tissues. (*B*) Boxplot of the sizes of promoter ACRs, nonpromoter ACRs, and SEs. (*C*) Distribution of distances to the closest genes of SEs. (*D*) DNase I sensitivity associated with constituent ACRs of SEs and other nonpromoter ACRs. DNase-seq data were developed from tissue harvested from 14-d seedlings. The DNase I sensitivity is normalized by size of the regions. (*E*) The expression levels of cognate genes associated with SEs and with other nonpromoter ACRs.

One of the key features of mammalian SEs is that they span a cluster of constituent enhancers and a large genomic region (16, 17). To search for potential SEs in *A. thaliana*, we used the published DNase-seq datasets generated from 17 diverse tissues/ecotypes and developed a comprehensive accessible chromatin map (*SI Appendix*, Table S1). We identified a total of 84,925 ACRs using F-seq (22) (see *Materials and Methods*). Approximately 37% of these ACRs overlapped with 500-bp regions upstream of a transcription start site (TSS), and so are named promoter ACRs. The remaining ACRs are defined as nonpromoter ACRs. The nonpromoter ACRs span an average of 395 bp, shorter than the average 594 bp for promoter ACRs. A subset of nonpromoter ACRs were found to form large clusters spanning a big genomic region (*SI Appendix*, Fig. S1*A*). For example, a 6.8-kb region on chromosome 4 contained a total of 13 independent ACRs derived from different DNase-seq data sets (Fig. 1*A*). The genomic regions flanking this ACR cluster include two well-studied genes related to floral and leaf development, *APETALA2* (*AP2*) (AT4G36920) and *SPATULA* (*SPT*) (AT4G36930) (23, 24). We arbitrarily defined the nonpromoter ACR clusters that were larger than 1.5 kb (the top 2.5%) as SEs. A total of 749 SEs were identified using this criterion (see *Materials and Methods*) (Dataset S1). These SEs have an average size of 2.1 kb, ranging from 1.5 to 10.6 kb (Fig. 1*B*), and contain an average of five ACRs, which are also named the “constituent ACRs” of SEs (Dataset S1). “Clusters of enhancers”, or clusters of ACRs, were reported previously in *A. thaliana* (12). A set of 94 genes in *A. thaliana* were found to be associated with a cluster of enhancers (12).

### Genomic Positions and Features Associated with SEs in *A. thaliana*.

Among the 749 SEs, only 99 (13%) are located within genes. The majority (75) of these genic SEs span multiple introns and/or exons. However, 22 SEs are located within a single intron (*SI Appendix*, Fig. S1*B*). These SE-containing introns have an average size of 1.9 kb, which is significantly larger than the average intron size (165 bp) in *A. thaliana*. Interestingly, two SEs were located within an exon of genes AT5G17980 and AT2G27660, respectively (*SI Appendix*, Fig. S1*C*). Both AT5G17980 and AT2G27660 are single-exon genes.

The remaining 650 SEs are located in intergenic regions. The majority (95%) of these intergenic SEs are within 3.2 kb of a TSS or a transcription termination site (TTS) of their closest gene (Fig. 1*C*). A few SEs are distant from any flanking genes. For example, a SE was found to be 4.8 kb away from the TSS of gene *GPAT6* (AT2G38110), which is important for tapetum development and fertility (26) (*SI Appendix*, Fig. S1*D*). Approximately half of the intergenic SEs are located to the 5′ end of at least one of the two flanking genes. However, 35 SEs are located to the 3′ ends of genes on both sides.

We next compared the DNase I sensitivity levels between the “constituent ACRs” within SEs and the rest of the nonpromoter ACRs. The constituent ACRs showed a higher level of DNase I sensitivity in all DNase-seq samples analyzed (Fig. 1*D*), which is similar to the high level of chromatin accessibility associated with SEs in mammalian species (16, 17). In addition, the SE-cognate genes (genes that are the closest to an SE, see *Materials and Methods*) showed a higher level of expression in most tissues compared with genes associated with other nonpromoter ACRs (Fig. 1*E* and *SI Appendix*, Table S2), which is consistent with the higher level of DNase I sensitivity associated with constituent ACRs within SEs.

### Chromatin Organization of SEs.

Interphase chromosomes form kilobase- to megabase-sized topologically associating domains (TADs). These TADs generate isolated gene regulatory domains in which enhancers may regulate multiple genes but their effects on genes in neighboring TADs are blocked (27). Although TADs are not a dominant chromatin feature in the *A. thaliana* genome, TAD-like structures can be identified in high-resolution 3D chromatin studies (21, 28, 29). Approximately 1,000 TAD-like regions were identified at 2-kb resolution (28).

**Fig. 2.**
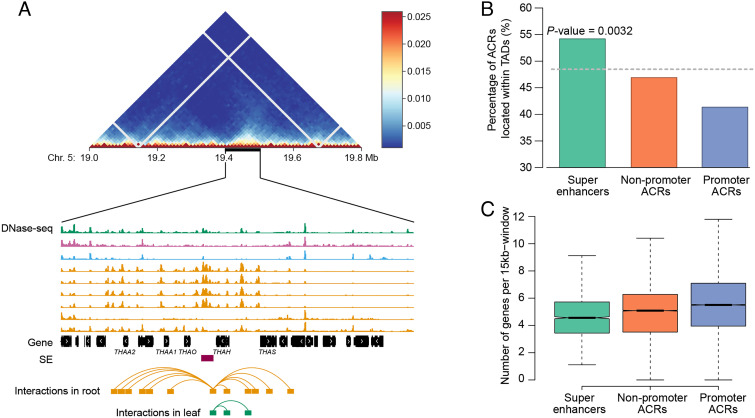
Chromatin organization of SEs. (*A*) A SE associated with the thalianol gene cluster that is located within a TAD. *Upper* panel shows the Hi-C map from 19.0 to 19.8 megabase on chromosome 5 at 10-kb resolution. The black bar marks the position of a TAD. *Middle* panel shows the DNase-seq tracks within the TAD. DNase-seq of 1-wk-old seedling (Col-0) (green), open flower (purple), seed coat cells (light blue), and root samples (orange) are included. *Lower* panel: chromatin loops between the SE and the thalianol genes in root and leaf are plotted based on Hi-C data sets. (*B*) Percentage of different classes of ACRs located within TADs. (*C*) Gene density of 15-kb windows containing different classes of ACRs.

We explored the association between these TADs and SEs. We first analyzed three well-characterized TADs that contain coregulated metabolic gene clusters (21). At least one SE was identified in each of these three TADs (*SI Appendix*, Fig. S3). For example, the thalianol gene cluster is located within a TAD spanning 19.4–19.5 megabases on chromosome 5 (Fig. 2*A*) and includes five genes: thalianol acyltransferase 2 (*THAA2*), thalianol acyltransferase 1 (*THAA1*), thalianol oxidase (*THAO*), thalianol hydroxylase (*THAH*) and thalianol synthase (*THAS*) (21, 30). An SE was identified in between the 3′ end of *THAO* and the 3′ end of *THAH*. This SE spans 3.6 kb and exhibits the highest level of chromatin accessibility in root tissue, which is in accordance with the fact that the thalianol genes are highly expressed in roots (31). Analysis of published Hi-C datasets (21) revealed multiple interactions between this SE and the thalianol genes in root tissue (Fig. 2*A*).

To test whether SEs are enriched within TADs, we calculated the percentages of SEs, promoter ACRs, and other nonpromoter ACRs that are located within TADs. We generated 1,000 random nongenic regions with the same sizes as the three types of ACRs to obtain the empirical distribution of percentage of regions located within TADs. We found that 49.7% of SEs are located within TADs (Dataset S1), significantly higher than for random nongenic regions (*P *= 0.003, empirical test). In contrast, promoter ACRs and other nonpromoter ACRs were not enriched in TADs (Fig. 2*B*). Enrichment of SEs in TAD-like chromatin domains in *A. thaliana* supports the hypothesis that an isolated chromatin domain covering an SE and the SE-cognate genes may facilitate precise regulation and block interference with/from neighboring genes and CREs (18).

SEs and their cognate genes tend to be located in gene desert regions in mammalian species (18). For example the *Sox2* gene, which is essential for pluripotency in mammals, and its SE are located within a 1.5-megabase gene desert (32). Location within low-gene density regions of the genome may facilitate the insulation of the SEs and their cognate genes. To test whether the SEs in *A. thaliana* are also located in regions with a relatively low gene density, we calculated the gene density of the *A. thaliana* genome using 15-kb sliding windows with 5-kb steps. Since the large sizes of SEs may impact the calculation of gene density, the lengths of SEs were subtracted in each window during gene density calculation (see *Materials and Methods*). We found that genomic regions containing SEs exhibited a significantly lower gene density than promoter ACRs and other nonpromoter ACRs (*P *< 2.2 × 10^−16^, one-sided Wilcoxon–Mann–Whitney test) (Fig. 2*C*).

### Contribution of SEs and Their Cognate Genes to Tissue Identity in *A. thaliana*.

**Fig. 3.**
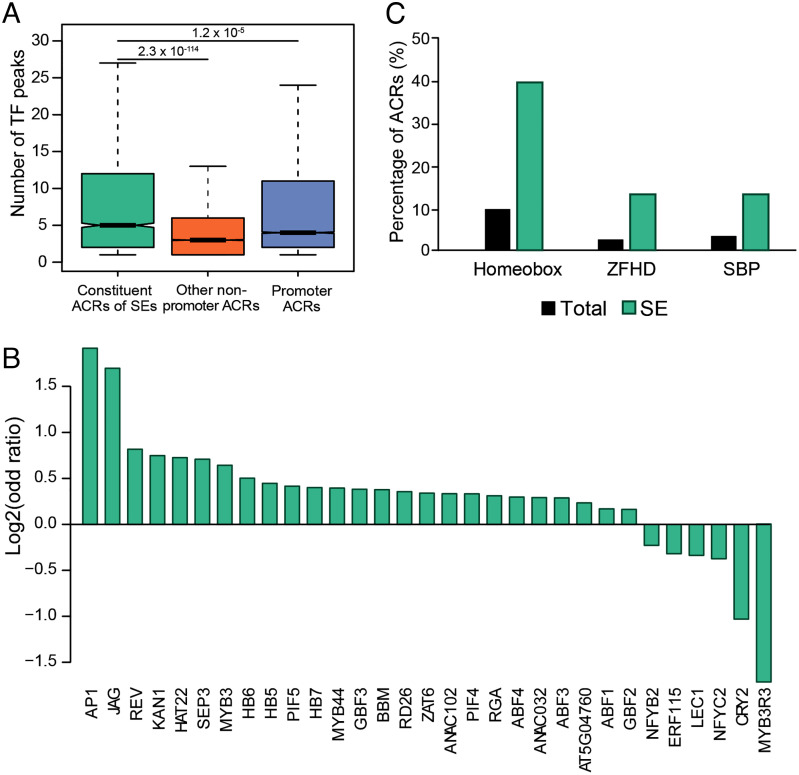
TFs associated with SEs. (*A*) TF numbers associated with the three classes of ACRs. The ChIP-seq peaks of 52 TFs were intersected with three classes of ACRs. For each class of ACRs, the number of TFs overlapped with each ACR was calculated and plotted as Boxplot. (*B*) Enrichment of TFs within constituent ACRs of SEs in comparison with promoter ACRs. (*C*) Enrichment of three TF families at constituent ACRs of SEs.

In mammalian species, SE-cognate genes identified in a specific cell type are highly enriched for the biological processes that define that cell type (3, 19). We, therefore, next explored the biological function of the SEs and their cognate genes in *A. thaliana*. We first examined whether the SEs are bound to specific TFs using published chromatin immunoprecipitation (ChIP)-seq data for 52 TFs in *A. thaliana* (33). We calculated the numbers of TFs bound to the constituent ACRs of SEs, promoter ACRs, and other nonpromoter ACRs. We found that the constituent ACRs were bound by the highest number (density) of TFs (Fig. 3*A*), and this was not due to the size of SEs or their distances from genes (*SI Appendix*, Fig. S4). We next asked if the binding sites of any TFs are enriched within the SEs. Enrichment was analyzed by calculating the log-odds ratio of each TF in pairwise comparisons among the three different classes of ACRs (see *Materials and Methods*). Several TFs were enriched within the constituent ACRs (Fig. 3*B*). Many of these TFs, such as APETALA1 (AP1) and JAGGED (JAG) (Fig. 3*B*), are key regulators of tissue development and organ identity. AP1 is involved in specifying the identity of floral meristem and regulates petal development (34). JAG plays an important role in controlling proper lateral organ shape of leaves, sepals, petals, and stamens (35).

We also analyzed the enrichment of the TF-binding motifs derived from DNA affinity purification sequencing (DAP-seq) in *A. thaliana* (36). Among the 529 TFs analyzed by DAP-seq, 47 were enriched within the constituent ACRs. The Homeobox, zinc finger homeodomain (ZFHD) and squamosa promoter binding protein (SBP) TF families were especially overrepresented (Fig. 3*C*). The Homeobox and ZFHD TF families are involved in meristem differentiation and organ identity (37, 38). The SBP TFs, including squamosa-promoter binding protein-like (SPL) 3, participate in floral development (39, 40).

We next used gene ontology (GO) enrichment to analyze the functions of the SE-cognate genes in *A. thaliana*. It has long been a challenge to accurately identify genes cognate to each enhancer. A recent study in mammalian species showed that distance-based methods perform adequately compared with the correlation-based approaches and machine-learning methods that rely on genomic and epigenomic datasets (41). Thus, we simply assigned the closest gene to each SE as the putative cognate gene. A total of 722 genes were assigned to the 749 SEs. Compared with promoter ACRs, SE-cognate genes were enriched with “biological processes”, including “regulation of molecular function” and “multicellular organism development” (*SI Appendix*, Table S3). SE-cognate genes were also enriched with the same GO terms when compared with genes associated with the other nonpromoter ACRs (*SI Appendix*, Table S3). Thus, the SE-cognate genes are distinctly enriched in functions related to development.

Collectively, these results showed that SEs as well as their cognate genes play a major role in organ development and tissue identity in *A. thaliana*, mirroring the functional characteristics of SEs and their cognate genes identified in mammalian species.

### Evolutionary Conservation of the *A. thaliana* SEs in Brassicaceae Species.

**Fig. 4.**
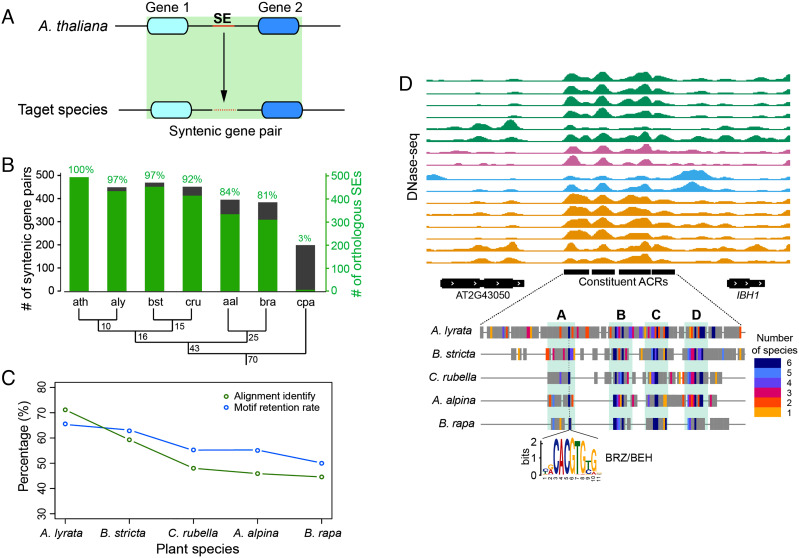
Evolution of SE-related DNA sequences in Brassicaceae species. (*A*) A scheme for identification of orthologous sequences of SEs in a species related to *A. thaliana*. (*B*) Conservation of SE-related DNA sequences in Brassicaceae species (ath: *A. thaliana*; aly: *A. lyrata*; bst: *Boechera stricta*; cru: *C. rubella*; aal: *Arabis alpina*; bra: *B. rapa*) and papaya (cpa: *Carica papaya*) within Brassicales. Syntenic gene pairs that span an SE in *A. thaliana* were identified in each species. Orthologous SEs were found in most syntenic gene pairs in all five Brassicaceae species. For example, 81% of the syntenic gene pairs in *B. rapa* included an orthologous SE. The gray boxes represent the numbers of synteny pairs, the green boxes represent the numbers of orthologous SEs. The percentages of synteny pairs containing orthologous SEs are labeled. The phylogenetic tree of the seven species is shown at the *Bottom* of the panel. The numbers in the phylogenetic tree (in millions of years) represent the divergence time of the branches*.* (*C*) Sequence identity and TF-binding motif retention rate of SEs among the five Brassicaceae species. Sequence identity is calculated as the percentage of each SE sequence in *A. thaliana* that can be aligned to a target species. TF motif retention rate is calculated as the percentage of TF-binding motifs identified in *A. thaliana* that also exists in the orthologous sequences in the five Brassicaceae species. (*D*) Conservation of TF-binding motifs within a SE located upstream of *IBH1*. The *Top* panel shows the DNase-seq tracks. The colors and order of the DNase-seq samples are the same in Fig. 1*A*. The four constituent ACRs in the SE are marked by black bars. The *Lower* panel shows the sequence alignment of the SE from five species. Positions of TF motifs are marked by colored bars. Number of species that contain the motifs were indicated by the color key. Gray color represents matched sequences with no motif identified. Lines represent no sequence alignment. A, B, C, and D marked four regions that contain conserved motifs. The sequence logo plot is the motif of BRZ/BEH, which regulates *IBH1* (41).

We were interested in the degree of conservation or divergence of the SE-related DNA sequences during evolution. We chose five Brassicaceae species that have different phylogenetic distances to *A. thaliana*, for our evolutionary study (Fig. 4*B*). To map the orthologous sequences of the SEs in the five species, we first identified syntenic gene pairs between *A. thaliana* and the five selected species (see *Materials and Methods*). The sequence of each SE was then aligned to the sequence between the two genes of a syntenic gene pair in a target species (Fig. 4*A*). We identified 17,364 syntenic gene pairs in at least one of the five species. Of the 749 SEs identified in *A. thaliana*, 498 (66.3%) were found to be located within a syntenic gene pair in at least one of the five Brassicaceae species. We reasoned that if the SEs identified in *A. thaliana* have an impact on transcription of their neighboring genes, we would expect strong synteny conservation of the gene pairs spanning SEs. To test this hypothesis, we calculated the number of species that maintained synteny of gene pairs spanning SEs. We found that 70% of the 498 gene pairs maintained synteny in at least five of the six species analyzed, which is significantly higher that gene pairs without associated SEs (50%, *P *< 1.0 × 10^−3^, empirical test).

We next asked how the SE sequences evolved during the evolution of the Brassicaceae species. To our surprise, most of the *A. thaliana* SEs are highly conserved among the Brassicaceae species. For example, 81% (312 of 384) of the *A. thaliana* SEs spanned by syntenic gene pairs showed homology to the orthologous sequences in *Brassica rapa*, which diverged from the common ancestor to *A. thaliana* approximately 43 Mya (43) (Fig. 4*B*). In contrast, only 48% of the promoters of the same syntenic genes showed homology to the promoters of the orthologous genes in *B. rapa* (*SI Appendix*, Fig. S5). The sequence identity (portion of matched nucleotides over aligned regions, see *Materials and Methods*) of the SEs did not change linearly with the phylogenetic distance (Fig. 4*C*). The sequence identity of SEs clearly dropped after about 16 My of divergence, reduced from 71% in *Arabidopsis lyrata* to 48% in *Capsella rubella* (Fig. 4*C*). However, the sequence identity of SEs remained at 46% and 45% in *Arabis alpina* and *B. rapa*, although these two species split from the common ancestor of *A. thaliana* about 43 Mya. The maintenance of sequence identity among distantly related species indicates that the ancestral DNA associated with the SEs may contain core regulatory sequences that are biologically significant and, therefore, have been maintained during evolution. To test this hypothesis, we identified all TF-binding motifs in the SEs of *A. thaliana* and calculated the motif retention rate based on the percentage of these motifs that also exists in the orthologous sequences in other Brassicaceae species (see *Materials and Methods*). The motif retention rate was more stable across the species compared with sequence identity (Fig. 4*C*). Indeed, 73% (363 of 498) of SEs were found to contain conserved noncoding sequences (CNSs) (44).

For example, an SE containing four constituent ACRs was found upstream of gene *ILI1 BINDING BHLH 1* (*IBH1*) (Fig. 4*D*) encoding a basic helix–loop–helix (bHLH) TF that regulates cell elongation via the brassinosteroid pathway (42, 45, 46). Four conserved sequence blocks (A, B, C, and D) were identified within the SE by multiple sequence alignment (Fig. 4*D*). Several conserved TF-binding motifs were identified within each conserved sequence block, including BRASSINAZOLE-RESISTANT 1 (BZR1), CYCLING DOF FACTOR 5 (CDF5), ETHYLENE RESPONSE FACTOR 11 (ERF11) and DNA BINDING WITH ONE FINGER 5.5 (DOF5.5). Of These TFs, BZR1, whose motif located within block A, is a known regulator of *IBH1* (42). Of note, only the sequence conservation of SEs within Brassicaceae was analyzed in this study. The function of the orthologous sequences – for example, chromatin accessibility and preservation of enhancer functionality – of SEs in other species is unknown. Thus further studies are required to decipher the functional conservation of SEs within Brassicaceae species.

### Genomic Disruption of a SE Associated with the Thalianol Gene Cluster.

**Fig. 5.**
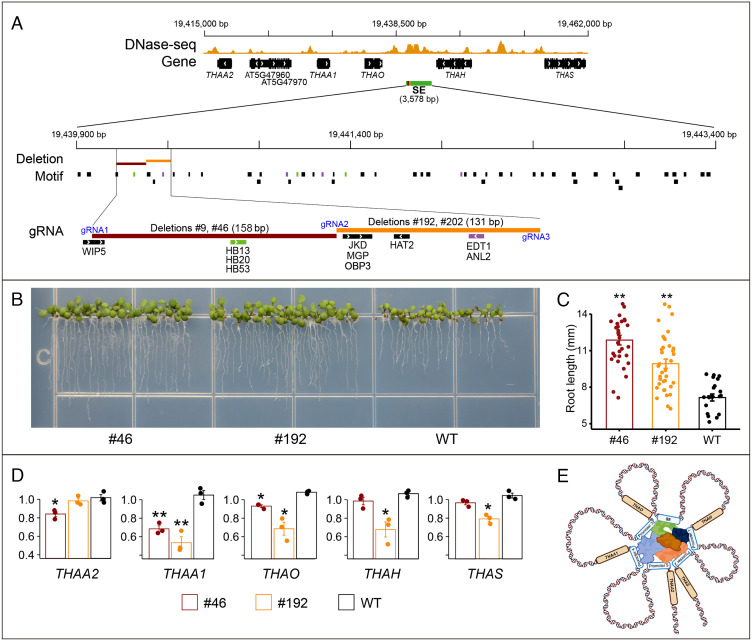
Functional validation of an SE associated with the thalianol gene cluster. (*A*) IGV tracks of the genomic region containing the thalianol gene cluster and a representative DNase-seq dataset derived from root tissue (SRR2101859). The SE is located between genes *THAO* and *THAH*. The positions of binding motifs for all TFs related to root development are marked. Three HB13-binding sites (green) and four EDT1-binding sites (purple) are marked by colors. Two small regions, marked in maroon and brown, respectively, were targeted for deletion. These two regions are also exemplified in detail. Three gRNAs used for CRISPR/Cas editing are also shown. (*B*) Phenotypes of deletion lines #192 and #46 at the 4-d seedling stage. (*C*) Measurements of primary root lengths of deletion lines #192 and #46 at the 4-d seedling stage. ** indicates significant *t* tests (*P* < 0.01). (*D*) The relative expression levels of the five thalianol genes in deletion lines #192 and #46 at the 4-d seedling stage. Significant *t* tests are marked by * (*P *< 0.05) or ** (*P *< 0.01). (*E*) A model of SE-mediated regulation of expression of the thalianol genes. The SE is proposed to interacts with all five promoters via transcriptional coactivators as well as TFs that are marked with different colors.

We selected the SE associated with the thalianol gene cluster for functional validation. Analysis of Hi-C datasets suggested that this SE interacts with multiple genes in root tissue (Fig. 2*A*). Thus, we predicted that modification of this SE would alter the pattern of the root-specific expression of multiple genes within the cluster. This SE (3,578 bp) contains putative binding sites for 21 different TFs that are known to be associated with root development (*SI Appendix*, Table S4). We attempted to delete a 278-bp region that showed the highest level of DNase I accessibility (in root tissue) within the SE (Fig. 5*A*). This region contains predicted binding sites for five TFs related to root development, including WIP DOMAIN PROTEIN 5 (WIP5), HOMEODOMAIN 13 (HB13), JACKDAW (JKD), HOMEOBOX ARABIDOPSIS THALIANA 2 (HAT2) and ENHANCED DROUGHT TOLERANCE 1 (EDT1) (47–52) (Fig. 5*A*). Most interestingly, HB13 and EDT1 are considered as negative regulators of root length (50, 51). Three guide RNAs (gRNAs) were designed for the development of two short deletions that span the HB13 and EDT1 binding sites, respectively (Fig. 5*A*).

We obtained two deletion lines (#9 and #46) that had lost a 158-bp region spanning the HB13 binding site, and a further two deletion lines (#192 and #202) lacking a 131-bp region spanning the EDT1 binding site (Fig. 5*A*). Interestingly, the deletion lines had longer primary roots compared with wild-type plants during early development (Fig. 5 *B* and *C*), a phenotype similar to those reported for mutations of the thalianol cluster genes (20) and also mimicking the phenotypes of *hb13* and *edt1* (50, 51). The phenotypes of the elongated primary roots are also correlated with losses of the binding sites of HB13 and EDT1, the two negative regulators for root length.

**Fig. 6.**
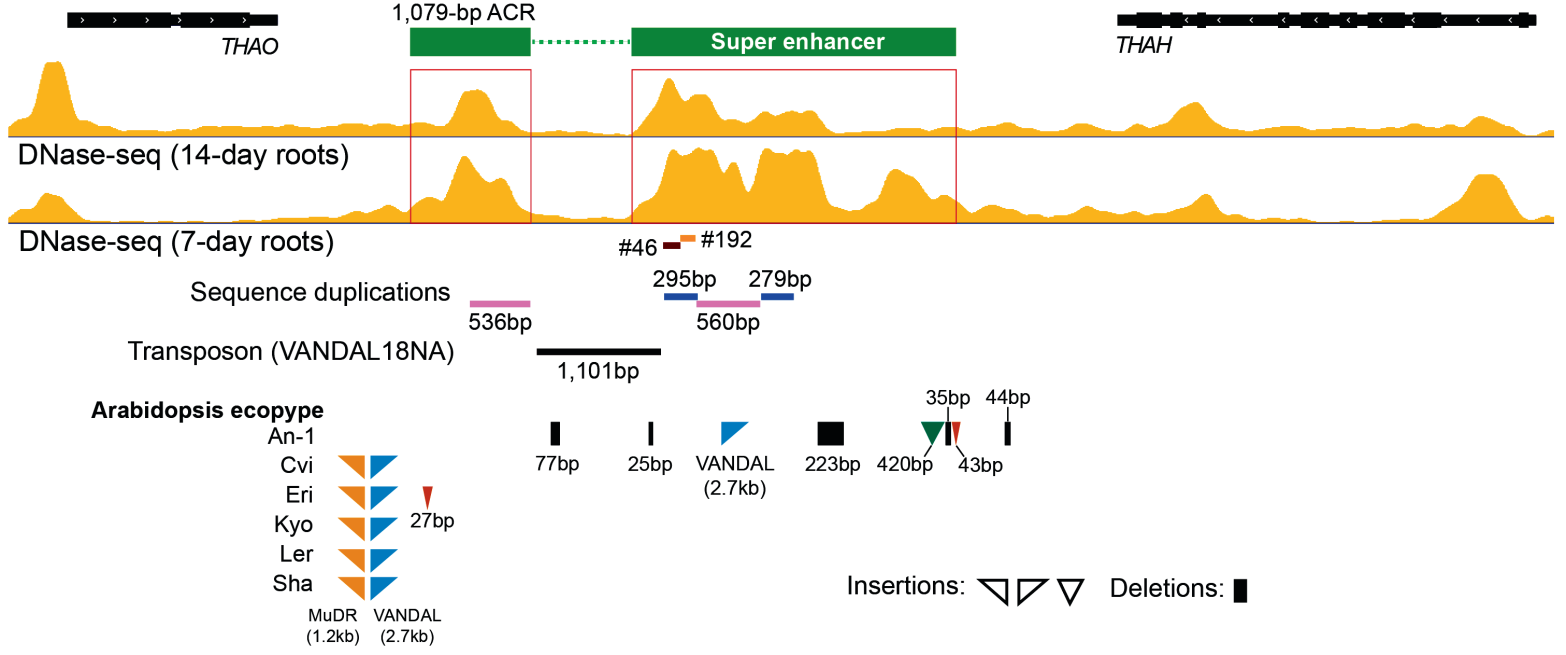
Sequence analysis of the SE associated with the thalianol gene cluster. Two duplications and a DNA transposon are identified within the SE. The 1,079 bp ACR should be part of the SE. Variation of the intergenic sequences between genes *THAO* and *THAH* is illustrated for six well-sequenced *A. thaliana* accessions. Most sequence variation was detected in this region from *A. thaliana* ecotype An-1. Only insertions/deletions larger than 20 bp were showed.

*THAO* and *THAA1*, the two genes upstream of the SE, became down-regulated in all deletion lines. In contrast, *THAH* and *THAS*, the two genes downstream of the SE, were down-regulated only in #192, which is closer to these two genes than deletion #46 (Fig. 5*D*). *THAA2*, which is the most distant gene from the SE, was down-regulated in #46 but not in #192. We also examined the expression of two intervening nonthalianol genes, AT5G47960 and AT5G47970, within the cluster (Fig. 5*A*). Interestingly, the transcription of both genes was significantly reduced in #46 (*SI Appendix*, Fig. S6). In contrast, gene AT5G45390, which is located outside of the TAD that spans the thalianol gene cluster, was not changed in the deletion lines (*SI Appendix*, Fig. S6). Sequence analysis revealed two small duplications in the SE region. A 295-bp region that spans the two CRISPR/Cas deletions shares 82% sequence similarity with a 279-bp downstream region within the SE (Fig. 6). Interestingly, a 560-bp region shares 86% sequence similarity with a 536-bp region located in a 1,079-bp ACR upstream of the SE (Fig. 6). This upstream ACR was separated from the SE by a 1,101-bp DNA transposon (VANDAL 18NA). This transposon is universally present in >1,000 sequenced *A. thaliana* accessions (53). Therefore, this ACR is likely part of the SE but was excluded computationally from our default SE identification system. The two small duplications would appear to be a single ~850-bp tandem duplication before the insertion of the transposon (Fig. 6). This duplication could have been induced by the transposition event. Several *A. thaliana* accessions have been sequenced with reference genome quality (54). Analysis of the intergenic sequences between genes *THAO* and *THAH* in these accessions revealed additional insertions of DNA transposons (MuDR and VANDAL) in this region, including an additional VANDAL copy in the middle of the SE in the An-1 ecotype (Fig. 6).

**Fig. 7.**
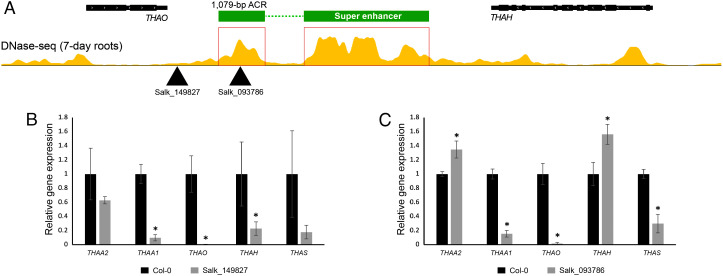
T-DNA insertions in the SE region and their impact on transcription of the five thalianol cluster genes. (*A*) Positions of T-DNA insertion of Salk_149827 and Salk_093786. (*B*) Gene expression analysis of the five thalianol cluster genes in root samples derived from T-DNA line Salk_149827 and wild-type Col-0 plants. (*C*) Gene expression analysis of the five thalianol cluster genes in root samples derived from T-DNA line Salk_093786 and wild-type Col-0 plants. Gene expression was measured by qRT-PCR and the Col-0 wild-type transcription level set as 1. PP2AA3 (At1g13320) was used as an internal control. Error bars indicate standard deviation of three biological replicates. Statistical significance (*t *test): *P < 0.05.

We identified two transfer DNA (T-DNA) lines inserted in the SE region. The T-DNA of SALK_149827 inserted around 194,36,952 bp on chromosome 5 (the exact position is not determined). This T-DNA is approximately 203 bp away from the TTS of gene *THAO* (Fig. 7*A*). The T-DNA of SALK_093786 inserted at 194,38,395 bp (exact position is determined). This T-DNA is located in the middle of the 1,079-bp ACR and is 1,646 bp away from the TTS of *THAO* (Fig. 7*A*). In both lines, transcription levels of the *THAO* gene were drastically reduced. Furthermore, significant transcriptional repression was observed for the *THAA1* and *THAH* genes in line SALK_149827 (Fig. 7*B*) and the *THAA1* and *THAS* genes in line SALK_093786. Remarkably, we also detected a significant upregulation for the genes *THAA2* and *THAH* in line SALK_093786 (Fig. 7*C*). The mis-regulation of the thalianol cluster genes in both lines with disrupted SE structure further supports the important role of the SE in cluster transcription. In contrast, insertion of T-DNA into individual thalianol cluster genes resulted in repression of the targeted gene only and had no impact to the rest of the genes in the cluster (20).

## Discussion

The “SE” concept was developed in mammalian species more than 10 y ago (3, 16, 17). The functional relevance of SEs, however, is still under discussion. There is some controversy about whether SEs have fundamentally different functions compared with regular enhancers (55, 56). Nevertheless, in addition to their large sizes, SEs in mammalian species have several features that distinguish them from regular enhancers, including their association with high levels of transcriptional coactivators, histone mark H3K27ac, and chromatin accessibility, and their recruitment of high concentration of TFs (17, 57). Most importantly, SEs have been recognized to regulate genes that have prominent roles in cell type identity and function in mammalian species (58). We characterized a set of the 749 largest ACR clusters in the *A. thaliana* genome. These SEs are preferentially associated with TAD-like chromatin domains. Our analyses also reveal that both the SEs and their cognate genes play roles in organ development and tissue identity in *A. thaliana.* Thus, the *A. thaliana* SEs mirror the functional characteristics associated with mammalian SEs.

Although the individual constituent enhancers within SEs have often been presumed to act synergistically (59), deletion mapping of several SEs in mammalian species suggest that individual enhancers within the clusters are usually functionally redundant (60) and can act in an additive manner (61, 62). Analysis of the SE associated with the thalianol gene cluster revealed a similar functional redundancy of the constituent enhancers. The duplicated regions within the SE showed more than 80% sequence homology and shared putative binding sites for TFs related to root development, suggesting that the duplicons have similar functions. However, in-depth deletion mapping will be required to confirm the functional redundancy of these duplicated sequences.

Rapid evolution of enhancers is a universal feature of mammalian genomes. Sequence analysis across 20 mammalian species showed that enhancers have evolved appreciably more rapid than promoters (63). However, SEs associated with developmentally essential genes can be highly conserved among distantly related mammalian species (59, 64). Nearly 42% of zebrafish SEs are located in close proximity to orthologs that are also associated with SEs in mouse and human despite the low overall SE sequence conservation (65). Therefore, the conservation of SEs, in contrast to the rapid evolution of normal enhancers, may be related to the conservation of biologically important CREs within the SEs (66, 67) and to the evolutionary stability of their cognate genes (65, 68). We demonstrate that the *A. thaliana* SEs show high-level conservation among the Brassicaceae species. These SEs are even more conserved than the corresponding promoters based on analysis of the syntenic gene pairs in different species (*SI Appendix*, Fig. S5). Thus, the *A. thaliana* SEs show a similar evolutionary pattern to those reported in animal species.

The raison d’etre for the existence of BGCs in plant genomes is intriguing. These clusters have features in common with bacterial operons (physical clustering and co-expression) but, unlike operons, each gene has its own promoter (69, 70). Analysis of chromosome conformation capture data has revealed that active BGCs are often confined within a TAD identified in the tissue where the cluster is expressed (21). By contrast, inactive BGCs are associated with heterochromatin in tissues where the cluster is not expressed (21). Coregulation is therefore likely achieved epigenetically within a large chromatin domain spanning an entire BGC. In the current study, we demonstrate that SEs may play a central role in the coregulation of these operon-like gene clusters. Deletions of a small portion of the SE can cause significant repression of all five genes of the thalianol cluster (Fig. 5*D*). The partial suppression of the genes observed in the deletion lines is likely due to the fact that the deletions (131–157 bp) account only a very small portion of the large SE as well as the presence of duplicated sequences related to the deleted DNA segment with the SE. It is worthy to note that additional intergenic ACRs are present in the thalianol gene cluster. Further dissection of these ACRs will be required to know if they play a role in the coregulation of the entire gene cluster, or in fine-tuning individual genes in the cluster.

Alteration of all five genes in the deletion lines suggests that the SE likely interacts with the promoters of all five genes (Fig. 5*E*). This hypothesis is supported by the fact that T-DNA insertions in the SE region can alter the transcription of all five thalianol cluster genes (Fig. 7). In contrast, T-DNA insertions into individual thalianol cluster genes only alter the expression of the target gene (20). The five thalianol genes have completely different promoter sequences. Interestingly, the SE and the five promoters share binding sites of many of the TFs related to root development (*SI Appendix*, Table S4). We have recently observed a similar phenomenon of shared TF binding sites between intronic enhancers and their cognate promoters in *A. thaliana* (15). Thus, the SE may interact with all five promoters via transcriptional co-activators as well as the common TFs (*SI Appendix*, Fig. 5E). We identified at least one in each of three other well-studied BGCs (*SI Appendix*, Fig. S3), suggesting that the SE-mediated system may be a common mechanism for the coregulation of biosynthetic gene clusters in plants.

## Materials and Methods

### Identification of ACRs and SEs.

A total of 17 DNase-seq datasets were obtained from NCBI Short Read Achieve. DNase-seq reads were mapped to *A. thaliana* TAIR10 genome using BWA aln (71) with default parameters and then convert to BAM format by SAMtools (72). Sequence reads with more than 20 mapping quality were retained for further analysis. ACRs were identified from each sample using F-seq (22). To determine the false discovery rate (FDR) for each sample, the same number of random reads as DNase-seq was generated 10,000 times and were called ACRs using F-seq. ACRs with FDR less than 0.001 were kept for further analysis. A union set of ACRs were generated by merging ACRs from all samples. These merged ACRs were classified into promoter ACRs, intergenic ACRs, and constituent ACRs of SEs. Promoter ACRs were defined as ACRs that overlap with 500 bp upstream of a TSS. The remaining nonpromoter ACRs were clustered if they are less than 50 bp apart. The top 2.5% of longest nonpromoter ACR clusters were defined as SEs. The individual ACR within each SE were defined as constituent ACRs. DNase I sensitivity of ACRs was calculated as the number of reads mapped to ACRs normalized by the total number of mapped reads in each sample. The genomic positions of three types of ACRs were determined by comparing the genomic coordinates of the ACRs to TAIR 10 gene annotation using BEDTools (73). To calculate gene density, genome was divided in to 15-kb windows with a step of 5 kb. Gene density for each window was calculated below:

$$\mathrm{Gene}\;\mathrm{density}=\frac{15,000\times \mathrm{number}\;\mathrm{of}\;\mathrm{genes}\;\mathrm{within}\;\mathrm{the}\;\mathrm{window}}{15\mathrm{kb}-\mathrm{total}\;\mathrm{length}\;\mathrm{of}\;\mathrm{ACRs}\;\mathrm{in}\;\mathrm{the}\;\mathrm{window}}.$$

### Chromatin Interaction Associated with SEs.

Hi-C data from leaves and roots (SRP224678) (21) were analyzed using FAN-C (74) with parameters “--restriction-enzyme BglII” for “fanc map” and “-l -us -m -p 1 -r BglII -q 20 –S” for “fanc pairs”. The interaction frequencies were normalized by HiCKRy from FitHiC2 (75) with the Knight–Ruiz algorithm. Chromatin loops were identified using FitHiC2 with parameters “-r 2,000 –m 5 –U 10,00,000.” Interactions with FDR less than 1 × 10^−10^ were retained for further analysis. One-sided Wilcoxon–Mann–Whitney test was conducted in R.

### TFs Occupancy and Binding Motif Analysis.

Peaks of the binding of 52 TFs were obtained from PlantPan3 (33). TF binding associated with three types of ACRs was analyzed using BEDTools. An ACR is bound by a TF if more than 50% of the ACR was covered by TF binding peaks. The odds ratio of each TF that bound constituent ACRs of SEs comparing with nonconstituent ACRs was calculated below:

$$\mathrm{Odds}\;\mathrm{ratio}=\frac{\frac{\mathrm{number}\;\mathrm{of}\;\mathrm{constituent}\;\mathrm{ACRs}\;\mathrm{bound}\;\mathrm{by}\;a\;\mathrm{TF}}{\mathrm{number}\;\mathrm{of}\;\mathrm{constituent}\;\mathrm{ACRs}\;\mathrm{not}\;\mathrm{bound}\;\mathrm{by}\;a\;\mathrm{TF}}}{\frac{\mathrm{number}\;\mathrm{of}\;\mathrm{non}\;\mathrm{constituent}\;\mathrm{ACRs}\;\mathrm{bound}\;\mathrm{by}\;a\;\mathrm{TF}}{\mathrm{number}\;\mathrm{of}\;\mathrm{non}\;\mathrm{constituent}\;\mathrm{ACRs}\;\mathrm{not}\;\mathrm{bound}\;\mathrm{by}\;\mathrm{TF}}}.$$

where non-constituent ACRs were promoter ACRs or intergenic ACRs. TF-binding motifs within ACRs were identified using FIMO (25) with default parameters against Plant Cistrome Database (36). TF motif retention rate is calculated as the percentage of TF-binding motifs identified in *A. thaliana* that also exists in the orthologous sequences in other Brassicaceae species. GO enrichment was conducted using goenrich (https://github.com/jdrudolph/goenrich). Conserved noncoding sequences (CNSs) were obtained from published report (44).

### Expression of Genes associated with SEs and Other Nonpromoter ACRs.

A gene that is the closest to an SE or to a nonpromoter ACR is defined as the gene cognate to the SE or to the nonpromoter ACR. Gene expression data were obtained from a previous study (76) and the TraVA database (travadb.org) (77, 78). The significance of difference of the expression levels between SE-cognate genes and nonpromoter ACR-cognate genes in each tissue was tested using one-sided Wilcoxon–Mann–Whitney test in R (4.0.4).

### Sequence Analysis and Evolution of SEs.

To analyze the sequence variation of the SE associated with the thalianol gene cluster, we mapped the VANDAL 18NA insertions in the 1,001 genome sequence database (53). The genomic sequencing data of 1,135 ecotypes were downloaded from NCBI Short Read Archive (SRP056687). Sequencing data from each ecotype were aligned to 1) left joint point (upstream 200 bp and 200 bp 5′ end of the VANDAL 18NA insertion), 2) right joint point (200 bp 3′ end of the VANDAL 18NA insertion and 200 bp downstream), and 3) cross joint (upstream 200 bp and downstream 200 bp of the VANDAL 18NA insertion). A VANDAL 18NA insertion was considered to be present if at least four read pairs span the left and right joint points but no read pair spans the cross joint. A VANDAL 18NA insertion was considered to be absent if at least four read pairs span the cross joint but no read pair spans the left or the right joint point.

Chromosome-level assemblies of seven *A. thaliana* ecotypes (An-1, C24, Cvi, Eri, Kyo, Ler, and Sha) were downloaded from project MPIPZJiao2020 (54). The genomic sequences from these ecotypes were aligned to the Col-0 genome (TAIR10) using minimap2 (79) with parameter “-a”. InDels were identified using BCFtools (80, 81) with parameters “-Q 0 -B -A -F 0 -m 0” for “bcftools mpileup” and “-cv –p 1” for “bcftools call”.

Syntenic gene between *A. thaliana* and other *Brassica* species (*A. lyrata, Boechera stricta*, *C. rubella*, *A. alpina*, and *B. rapa*) were identified using SynMap2 (82). Pairs of genes that upstream and downstream of a SE in *A. thaliana* were extracted. Syntenic gene pairs were defined as gene pairs that are located within synteny blocks in any one of the species were retained. To identify orthologous sequences of SEs in other *Brassica* species, we required that the syntenic gene pairs in target species are also adjacent pairs without insertion of any genes between them. SEs in *A. thaliana* were aligned to the sequences between the genes of syntenic gene pairs in target species using BLAT with parameter “-minIdentity = 70”. Motifs within SE orthologous sequences were identified using FIMO (25) with default parameters against Plant Cistrome Database (36). Conserved noncoding sequences (CNSs) were obtained from published study (44). Sequence identity is calculated as the percentage of matched nucleotides over the aligned region.

### Development and Characterization of Deletion Lines Using CRISPR/Cas.

Seeds of *A. thaliana* ecotype Col-0 were germinated on solid MS plates and transferred to soil and grown under normal greenhouse conditions (18–22°C, 16/8 h. light/dark, light intensity of 70 µmol m−2 s−1). Guide RNAs (gRNAs) were designed by CRISPR-GE (83) (*SI Appendix*, Table S6). The three gRNA expression cassettes driven by AtU6-26 were tandemly inserted into CAMBIA1300-pYAO:Cas9 vector through *Spe*I/*Nhe*I digestion (84). Homozygous deletion lines were generated by transforming wild-type *A. thaliana* (Col-0) plants with *A. tumefaciens* GV3101 via floral dipping as previously described (15). All homozygous deletion lines were finally confirmed by PCR using primers flanking the region (F-5′CTTCGAGAAGATAATGATGTATGG3′; R-5′TAAAAATGCGGTAGGTGCTAAC3′).

To quantify the primary root length, seeds of the homozygous deletion liens and wild-type Col-0 were germinated on square Petri dishes containing solid MS and kept in darkness at 4°C. The plates were transferred to a growth chamber with 16-h d and 8-h night at 22°C and positioned vertically. The 4-d-old seedlings were imaged by cannon 6D Mark II digital camera (Cannon, Japan) and were measured for their primary root length using Image J (85). Total RNAs were extracted from roots harvested from 4-d-old seedlings using the RNeasy Plant Mini Kit (Qiagen, Cat. # 74904). cDNA was synthesized using PrimeScript™ RT reagent Kit with gDNA Eraser (Takara, Cat. # RR047A). Quantitative real-time PCR (qPCR) was performed with gene-specific primers (*SI Appendix*, Table S7) using a total volume of 20 μl of TB Green® Fast qPCR Mix (Takara, Cat. # RR430A) on a LightCycler 480 (Roche) system (Roche, Switzerland). AtUBC21 was used as a reference gene. Experiments were taken for three technical replicates in three biological replicates.

## Supplementary Material

Appendix 01 (PDF)

Dataset S01 (XLSX)

Dataset S02 (XLSX)

## Data Availability

All study data are included in the article and/or *SI Appendix.*

## References

[1] D. Shlyueva, G. Stampfel, A. Stark, Transcriptional enhancers: From properties to genome-wide predictions. Nat. Rev. Genet. **15**, 272–286 (2014). 24614317 10.1038/nrg3682

[2] D. Lee, R. Karchin, M. A. Beer, Discriminative prediction of mammalian enhancers from DNA sequence. Genome Res. **21**, 2167–2180 (2011). 21875935 10.1101/gr.121905.111 PMC3227105

[3] D. Hnisz , Super-enhancers in the control of cell identity and disease. Cell **155**, 934–947 (2013). 24119843 10.1016/j.cell.2013.09.053 PMC3841062

[4] B. Zhu, W. L. Zhang, T. Zhang, B. Liu, J. M. Jiang, Genome-wide prediction and validation of intergenic enhancers in Arabidopsis using open chromatin signatures. Plant Cell **27**, 2415–2426 (2015). 26373455 10.1105/tpc.15.00537 PMC4815101

[5] H. N. Zhao , Genome-wide MNase hypersensitivity assay unveils distinct classes of open chromatin associated with H3K27me3 and DNA methylation in *Arabidopsis thaliana*. Genome Biol. **21**, 24 (2020). 32014062 10.1186/s13059-020-1927-5 PMC6996174

[6] N. D. Heintzman , Histone modifications at human enhancers reflect global cell-type-specific gene expression. Nature **459**, 108–112 (2009). 19295514 10.1038/nature07829 PMC2910248

[7] R. R. Catarino, A. Stark, Assessing sufficiency and necessity of enhancer activities for gene expression and the mechanisms of transcription activation. Genes Dev. **32**, 202–223 (2018). 29491135 10.1101/gad.310367.117 PMC5859963

[8] M. P. Creyghton , Histone H3K27ac separates active from poised enhancers and predicts developmental state. Proc. Natl. Acad. Sci. U.S.A. **107**, 21931–21936 (2010). 21106759 10.1073/pnas.1016071107 PMC3003124

[9] S. Smemo , Obesity-associated variants within *FTO* form long-range functional connections with *IRX3*. Nature **507**, 371–375 (2014). 24646999 10.1038/nature13138 PMC4113484

[10] R. Oka , Genome-wide mapping of transcriptional enhancer candidates using DNA and chromatin features in maize. Genome Biol. **18**, 137 (2017). 28732548 10.1186/s13059-017-1273-4 PMC5522596

[11] Z. F. Lu , The prevalence, evolution and chromatin signatures of plant regulatory elements. Nat. Plants **5**, 1250–1259 (2019). 31740772 10.1038/s41477-019-0548-z

[12] W. H. Yan , Dynamic control of enhancer activity drives stage-specific gene expression during flower morphogenesis. Nat. Commun. **10**, 1705 (2019). 30979870 10.1038/s41467-019-09513-2 PMC6461659

[13] Y. L. Xie , Enhancer transcription detected in the nascent transcriptomic landscape of bread wheat. Genome Biol. **23**, 109 (2022). 35501845 10.1186/s13059-022-02675-1 PMC9063354

[14] H. N. Zhao , Proliferation of regulatory DNA elements derived from transposable elements in the maize genome. Plant Physiol. **176**, 2789–2803 (2018). 29463772 10.1104/pp.17.01467 PMC5884613

[15] F. L. Meng , Genomic editing of intronic enhancers unveils their role in fine-tuning tissue-specific gene expression in *Arabidopsis thaliana*. Plant Cell **33**, 1997–2014 (2021). 33764459 10.1093/plcell/koab093 PMC8290289

[16] J. Loven , Selective inhibition of tumor oncogenes by disruption of super-enhancers. Cell **153**, 320–334 (2013). 23582323 10.1016/j.cell.2013.03.036 PMC3760967

[17] W. A. Whyte , Master transcription factors and mediator establish super-enhancers at key cell identity genes. Cell **153**, 307–319 (2013). 23582322 10.1016/j.cell.2013.03.035 PMC3653129

[18] X. Wang, M. J. Cairns, J. Yan, Super-enhancers in transcriptional regulation and genome organization. Nucleic Acids Res. **47**, 11481–11496 (2019). 31724731 10.1093/nar/gkz1038 PMC7145697

[19] S. C. J. Parker , Chromatin stretch enhancer states drive cell-specific gene regulation and harbor human disease risk variants. Proc. Natl. Acad. Sci. U.S.A. **110**, 17921–17926 (2013). 24127591 10.1073/pnas.1317023110 PMC3816444

[20] B. Field, A. E. Osbourn, Metabolic diversification - Independent assembly of operon-like gene clusters in different plants. Science **320**, 543–547 (2008). 18356490 10.1126/science.1154990

[21] H. W. Nutzmann , Active and repressed biosynthetic gene clusters have distinct chromosome states. Proc. Natl. Acad. Sci. U.S.A. **117**, 13800–13809 (2020). 32493747 10.1073/pnas.1920474117 PMC7306824

[22] A. P. Boyle, J. Guinney, G. E. Crawford, T. S. Furey, F-Seq: A feature density estimator for high-throughput sequence tags. Bioinformatics **24**, 2537–2538 (2008). 18784119 10.1093/bioinformatics/btn480 PMC2732284

[23] J. K. Okamuro, B. Caster, R. Villarroel, M. VanMontagu, K. D. Jofuku, The AP2 domain of APETALA2 defines a large new family of DNA binding proteins in Arabidopsis. Proc. Natl. Acad. Sci. U.S.A. **94**, 7076–7081 (1997). 9192694 10.1073/pnas.94.13.7076 PMC21287

[24] Y. Ichihashi, G. Horiguchi, S. Gleissberg, H. Tsukaya, The bHLH transcription factor *SPATULA* controls final leaf size in *Arabidopsis thaliana*. Plant Cell Physiol. **51**, 252–261 (2010). 20040585 10.1093/pcp/pcp184

[25] C. E. Grant, T. L. Bailey, W. S. Noble, FIMO: Scanning for occurrences of a given motif. Bioinformatics **27**, 1017–1018 (2011). 21330290 10.1093/bioinformatics/btr064 PMC3065696

[26] X. C. Li , Glycerol-3-phosphate Acyltransferase 6 (GPAT6) is important for tapetum development in *Arabidopsis* and plays multiple roles in plant fertility. Mol. Plant **5**, 131–142 (2012). 21746699 10.1093/mp/ssr057

[27] J. R. Dixon, D. U. Gorkin, B. Ren, Chromatin domains: The unit of chromosome organization. Mol. Cell **62**, 668–680 (2016). 27259200 10.1016/j.molcel.2016.05.018 PMC5371509

[28] C. M. Wang , Genome-wide analysis of local chromatin packing In *Arabidopsis thaliana*. Genome Res. **25**, 246–256 (2015). 25367294 10.1101/gr.170332.113 PMC4315298

[29] L. H. Sun , Heat stress-induced transposon activation correlates with 3D chromatin organization rearrangement in *Arabidopsis*. Nat. Commun. **11**, 1886 (2020). 32312999 10.1038/s41467-020-15809-5 PMC7170881

[30] Z. H. Liu , Formation and diversification of a paradigm biosynthetic gene cluster in plants. Nat. Commun. **11**, 5354 (2020). 33097700 10.1038/s41467-020-19153-6 PMC7584637

[31] A. C. C. Huang , A specialized metabolic network selectively modulates *Arabidopsis* root microbiota. Science **364**, 546 (2019). 10.1126/science.aau6389 31073042

[32] H. Y. Zhou , A *Sox2* distal enhancer cluster regulates embryonic stem cell differentiation potential. Genes Dev. **28**, 2699–2711 (2014). 25512558 10.1101/gad.248526.114 PMC4265674

[33] C. N. Chow , PlantPAN3.0: A new and updated resource for reconstructing transcriptional regulatory networks from ChIP-seq experiments in plants. Nucleic Acids Res. **47**, D1155–D1163 (2019). 30395277 10.1093/nar/gky1081 PMC6323957

[34] M. Ng, M. F. Yanofsky, Activation of the Arabidopsis B class homeotic genes by *APETALA1*. Plant Cell **13**, 739–753 (2001). 11283333 10.1105/tpc.13.4.739 PMC135542

[35] J. R. Dinneny, R. Yadegari, R. L. Fischer, M. F. Yanofsky, D. Weigel, The role of *JAGGED* in shaping lateral organs. Development **131**, 1101–1110 (2004). 14973282 10.1242/dev.00949

[36] R. C. O’Malley , Cistrome and epicistrome features shape the regulatory DNA landscape. Cell **165**, 1280–1292 (2016). 27203113 10.1016/j.cell.2016.04.038 PMC4907330

[37] Q. K. G. Tan, V. F. Irish, The *Arabidopsis* zinc finger-homeodomain genes encode proteins with unique biochemical properties that are coordinately expressed during floral development. Plant Physiol. **140**, 1095–1108 (2006). 16428600 10.1104/pp.105.070565 PMC1400567

[38] F. D. Ariel, P. A. Manavella, C. A. Dezar, R. L. Chan, The true story of the HD-Zip family. Trends Plant Sci. **12**, 419–426 (2007). 17698401 10.1016/j.tplants.2007.08.003

[39] G. H. Cardon, S. Hohmann, K. Nettesheim, H. Saedler, P. Huijser, Functional analysis of the *Arabidopsis thaliana* SBP-box gene SPL3: A novel gene involved in the floral transition. Plant J. **12**, 367–377 (1997). 9301089 10.1046/j.1365-313x.1997.12020367.x

[40] G. Cardon , Molecular characterisation of the *Arabidopsis* SBP-box genes. Gene **237**, 91–104 (1999). 10524240 10.1016/s0378-1119(99)00308-x

[41] J. E. Moore, H. E. Pratt, M. J. Purcaro, Z. P. Weng, A curated benchmark of enhancer-gene interactions for evaluating enhancer-target gene prediction methods. Genome Biol. **21**, 17 (2020). 31969180 10.1186/s13059-019-1924-8 PMC6977301

[42] L. Y. Zhang , Antagonistic HLH/bHLH transcription factors mediate brassinosteroid regulation of cell elongation and plant development in rice and *Arabidopsis*. Plant Cell **21**, 3767–3780 (2009). 20009022 10.1105/tpc.109.070441 PMC2814508

[43] M. A. Beilstein, N. S. Nagalingum, M. D. Clements, S. R. Manchester, S. Mathews, Dated molecular phylogenies indicate a Miocene origin for *Arabidopsis thaliana*. Proc. Natl. Acad. Sci. U.S.A. **107**, 18724–18728 (2010). 20921408 10.1073/pnas.0909766107 PMC2973009

[44] A. Haudry , An atlas of over 90,000 conserved noncoding sequences provides insight into crucifer regulatory regions. Nat. Genet. **45**, 891–898 (2013). 23817568 10.1038/ng.2684

[45] M. Ikeda, S. Fujiwara, N. Mitsuda, M. Ohme-Takagi, A triantagonistic basic helix-loop-helix system regulates cell elongation in *Arabidopsis*. Plant Cell **24**, 4483–4497 (2012). 23161888 10.1105/tpc.112.105023 PMC3531847

[46] M. K. Zhiponova , Helix-loop-helix/basic helix-loop-helix transcription factor network represses cell elongation in *Arabidopsis* through an apparent incoherent feed-forward loop. Proc. Natl. Acad. Sci. U.S.A. **111**, 2824–2829 (2014). 24505057 10.1073/pnas.1400203111 PMC3932932

[47] B. C. W. Crawford , Genetic control of distal stem cell fate within root and embryonic meristems. Science **347**, 655–659 (2015). 25612610 10.1126/science.aaa0196

[48] D. Welch , Arabidopsis JACKDAW and MAGPIE zinc finger proteins delimit asymmetric cell division and stabilize tissue boundaries by restricting SHORT-ROOT action. Genes Dev. **21**, 2196–2204 (2007). 17785527 10.1101/gad.440307 PMC1950858

[49] S. Sawa , The *HAT2* gene, a member of the HD-Zip gene family, isolated as an auxin inducible gene by DNA microarray screening, affects auxin response in *Arabidopsis*. Plant J. **32**, 1011–1022 (2002). 12492842 10.1046/j.1365-313x.2002.01488.x

[50] A. T. Silva, P. A. Ribone, R. L. Chan, W. Ligterink, H. W. M. Hilhorst, A predictive coexpression network identifies novel genes controlling the seed-to-seedling phase transition in *Arabidopsis thaliana*. Plant Physiol. **170**, 2218–2231 (2016). 26888061 10.1104/pp.15.01704 PMC4825141

[51] H. Yu , Activated expression of an Arabidopsis HD-START protein confers drought tolerance with improved root system and reduced stomatal density. Plant Cell **20**, 1134–1151 (2008). 18451323 10.1105/tpc.108.058263 PMC2390749

[52] M. Nakamura , Characterization of the class IV homeodomain-leucine zipper gene family in Arabidopsis. Plant Physiol. **141**, 1363–1375 (2006). 16778018 10.1104/pp.106.077388 PMC1533922

[53] C. Alonso-Blanco , 1,135 genomes reveal the global pattern of polymorphism in Arabidopsis thaliana. Cell **166**, 481–491 (2016). 27293186 10.1016/j.cell.2016.05.063 PMC4949382

[54] W. B. Jiao, K. Schneeberger, Chromosome-level assemblies of multiple Arabidopsis genomes reveal hotspots of rearrangements with altered evolutionary dynamics. Nat. Commun. **11**, 989 (2020). 32080174 10.1038/s41467-020-14779-y PMC7033125

[55] S. Pott, J. D. Lieb, What are super-enhancers? Nat. Genet. **47**, 8–12 (2015). 25547603 10.1038/ng.3167

[56] G. A. Blobel, D. R. Higgs, J. A. Mitchell, D. Notani, R. A. Young, Testing the super-enhancer concept. Nat. Rev. Genet. **22**, 749–755 (2021), 10.1038/s41576-021-00398-w. 34480110

[57] A. Khan, X. G. Zhang, dbSUPER: A database of super-enhancers in mouse and human genome. Nucleic Acids Res. **44**, D164–D171 (2016). 26438538 10.1093/nar/gkv1002 PMC4702767

[58] S. Heinz, C. E. Romanoski, C. Benner, C. K. Glass, The selection and function of cell type-specific enhancers. Nat. Rev. Mol. Cell Bio. **16**, 144–154 (2015). 25650801 10.1038/nrm3949 PMC4517609

[59] V. Honnell , Identification of a modular super-enhancer in murine retinal development. Nat. Commun. **13**, 253 (2022). 35017532 10.1038/s41467-021-27924-y PMC8752785

[60] S. D. Moorthy , Enhancers and super-enhancers have an equivalent regulatory role in embryonic stem cells through regulation of single or multiple genes. Genome Res. **27**, 246–258 (2017). 27895109 10.1101/gr.210930.116 PMC5287230

[61] A. J. Will , Composition and dosage of a multipartite enhancer cluster control developmental expression of Ihh (Indian hedgehog). Nat. Genet. **49**, 1539–1545 (2017). 28846100 10.1038/ng.3939 PMC5617800

[62] S. Q. Xie, J. L. Duan, B. X. Li, P. Zhou, G. C. Hon, Multiplexed engineering and analysis of combinatorial enhancer activity in single cells. Mol. Cell **66**, 285–299 (2017). 28416141 10.1016/j.molcel.2017.03.007

[63] D. Villar , Enhancer evolution across 20 mammalian species. Cell **160**, 554–566 (2015). 25635462 10.1016/j.cell.2015.01.006 PMC4313353

[64] J. Ryu, H. Kim, D. Yang, A. J. Lee, I. Jung, A new class of constitutively active super-enhancers is associated with fast recovery of 3D chromatin loops. BMC Bioinformatics **20**, 127 (2019). 30925856 10.1186/s12859-019-2646-3 PMC6439976

[65] Y. A. Perez-Rico , Comparative analyses of super-enhancers reveal conserved elements in vertebrate genomes. Genome Res. **27**, 259–268 (2017). 27965291 10.1101/gr.203679.115 PMC5287231

[66] J. M. Muino , Evolution of DNA-binding sites of a floral master regulatory transcription factor. Mol. Biol. Evol. **33**, 185–200 (2016). 26429922 10.1093/molbev/msv210 PMC4693976

[67] E. S. Wong , Deep conservation of the enhancer regulatory code in animals. Science **370**, 681 (2020). 10.1126/science.aax8137 33154111

[68] C. Berthelot, D. Villar, J. E. Horvath, D. T. Odom, P. Flicek, Complexity and conservation of regulatory landscapes underlie evolutionary resilience of mammalian gene expression. Nat. Ecol. Evol. **2**, 152–163 (2018). 29180706 10.1038/s41559-017-0377-2 PMC5733139

[69] A. Osbourn, Secondary metabolic gene clusters: Evolutionary toolkits for chemical innovation. Trends Genet. **26**, 449–457 (2010). 20739089 10.1016/j.tig.2010.07.001

[70] H. W. Nutzmann, C. Scazzocchio, A. Osbourn, Metabolic gene clusters in eukaryotes. Ann. Rev. Genet. **52**, 159–183 (2018). 30183405 10.1146/annurev-genet-120417-031237

[71] H. Li, R. Durbin, Fast and accurate short read alignment with Burrows-Wheeler transform. Bioinformatics **25**, 1754–1760 (2009). 19451168 10.1093/bioinformatics/btp324 PMC2705234

[72] H. Li , The sequence alignment/map format and SAMtools. Bioinformatics **25**, 2078–2079 (2009). 19505943 10.1093/bioinformatics/btp352 PMC2723002

[73] A. R. Quinlan, I. M. Hall, BEDTools: A flexible suite of utilities for comparing genomic features. Bioinformatics **26**, 841–842 (2010). 20110278 10.1093/bioinformatics/btq033 PMC2832824

[74] K. Kruse, C. B. Hug, J. M. Vaquerizas, FAN-C: A feature-rich framework for the analysis and visualisation of chromosome conformation capture data. Genome Biol. **21**, 303 (2020). 33334380 10.1186/s13059-020-02215-9 PMC7745377

[75] A. Kaul, S. Bhattacharyya, F. Ay, Identifying statistically significant chromatin contacts from Hi-C data with FitHiC2. Nat. Protoc. **15**, 991–1012 (2020). 31980751 10.1038/s41596-019-0273-0 PMC7451401

[76] W. L. Zhang, T. Zhang, Y. F. Wu, J. M. Jiang, Genome-wide identification of regulatory DNA elements and protein-binding footprints using signatures of open chromatin in *Arabidopsis*. Plant Cell **24**, 2719–2731 (2012). 22773751 10.1105/tpc.112.098061 PMC3426110

[77] A. V. Klepikova, M. D. Logacheva, S. E. Dmitriev, A. A. Penin, RNA-seq analysis of an apical meristem time series reveals a critical point in Arabidopsis thaliana flower initiation. BMC Genomics **16**, 466 (2015). 26084880 10.1186/s12864-015-1688-9 PMC4470339

[78] A. V. Klepikova, A. S. Kasianov, E. S. Gerasimov, M. D. Logacheva, A. A. Penin, A high resolution map of the *Arabidopsis thaliana* developmental transcriptome based on RNA-seq profiling. Plant J. **88**, 1058–1070 (2016). 27549386 10.1111/tpj.13312

[79] H. Li, New strategies to improve minimap2 alignment accuracy. Bioinformatics **37**, 4572–4574 (2021). 34623391 10.1093/bioinformatics/btab705 PMC8652018

[80] H. Li, A statistical framework for SNP calling, mutation discovery, association mapping and population genetical parameter estimation from sequencing data. Bioinformatics **27**, 2987–2993 (2011). 21903627 10.1093/bioinformatics/btr509 PMC3198575

[81] P. Danecek , Twelve years of SAMtools and BCFtools. Gigascience **10**, giab008 (2021). 33590861 10.1093/gigascience/giab008 PMC7931819

[82] A. Haug-Baltzell, S. A. Stephens, S. Davey, C. E. Scheidegger, E. Lyons, SynMap2 and SynMap3D: Web-based whole-genome synteny browsers. Bioinformatics **33**, 2197–2198 (2017). 28334338 10.1093/bioinformatics/btx144

[83] X. R. Xie , CRISPR-GE: A convenient software toolkit for CRISPR-based genome editing. Mol. Plant **10**, 1246–1249 (2017). 28624544 10.1016/j.molp.2017.06.004

[84] L. H. Yan , High-efficiency genome editing in Arabidopsis using YAO promoter-driven CRISPR/Cas9 system. Mol. Plant **8**, 1820–1823 (2015). 26524930 10.1016/j.molp.2015.10.004

[85] C. A. Schneider, W. S. Rasband, K. W. Eliceiri, NIH Image to ImageJ: 25 years of image analysis. Nat. Methods **9**, 671–675 (2012). 22930834 10.1038/nmeth.2089 PMC5554542

