# Supplementary material for: Identification and functional validation of super-enhancers in Arabidopsis thaliana
Source: Proc Natl Acad Sci U S A. 2022 Nov 21;119(48):e2215328119. doi: 10.1073/pnas.2215328119 (PMC9860255; doi:10.1073/pnas.2215328119)
Supplement: Supplementary file 1 — Appendix 01 (PDF) [file pnas.2215328119.sapp.pdf]

Supplemental Information

Identification and functional validation of super-enhancers in *Arabidopsis thaliana*

Hainan Zhao[a,1], Mingyu Yang[b,1], Jade Bishop[c], Yuhan Teng[b], Yingxue Cao[a,b], Brandon D. Beall[a], Shuanglin Li[b], Tongxin Liu[b], Qingxi Fang[b], Chao Fang[a], Haoyang Xin[a], Hans-Wilhelm Nützmann[c], Anne Osbourn[d,2], Fanli Meng[b,e,2], and Jiming Jiang[a,f,g,2]

[a] Department of Plant Biology, Michigan State University, East Lansing, MI 48824, USA

[b] Key Laboratory of Soybean Biology in Chinese Ministry of Education, Northeast Agricultural University, Harbin 150030, China

[c] The Milner Centre for Evolution, Department of Biology and Biochemistry, University of Bath, Bath BA2 7AY, UK

[d] Department of Biochemistry and Metabolism, John Innes Centre, Norwich Research Park, Colney Lane, Norwich, NR4 7UH, UK

[e] Northeast Institute of Geography and Agroecology, Key Laboratory of Soybean Molecular Design Breeding, Chinese Academy of Sciences, Harbin 150081, China

[f] Department of Horticulture, Michigan State University, East Lansing, MI 48824, USA

[g] Michigan State University AgBioResearch, East Lansing, MI 48824, USA

[1] These authors contribute to this work equally

[2] To whom correspondence may be addressed. Email: anne.osbourn@jic.ac.uk, mengfanli@neau.edu.cn or jiangjm@msu.edu

This PDF file includes: Figures S1 to S6; Tables S1 to S7

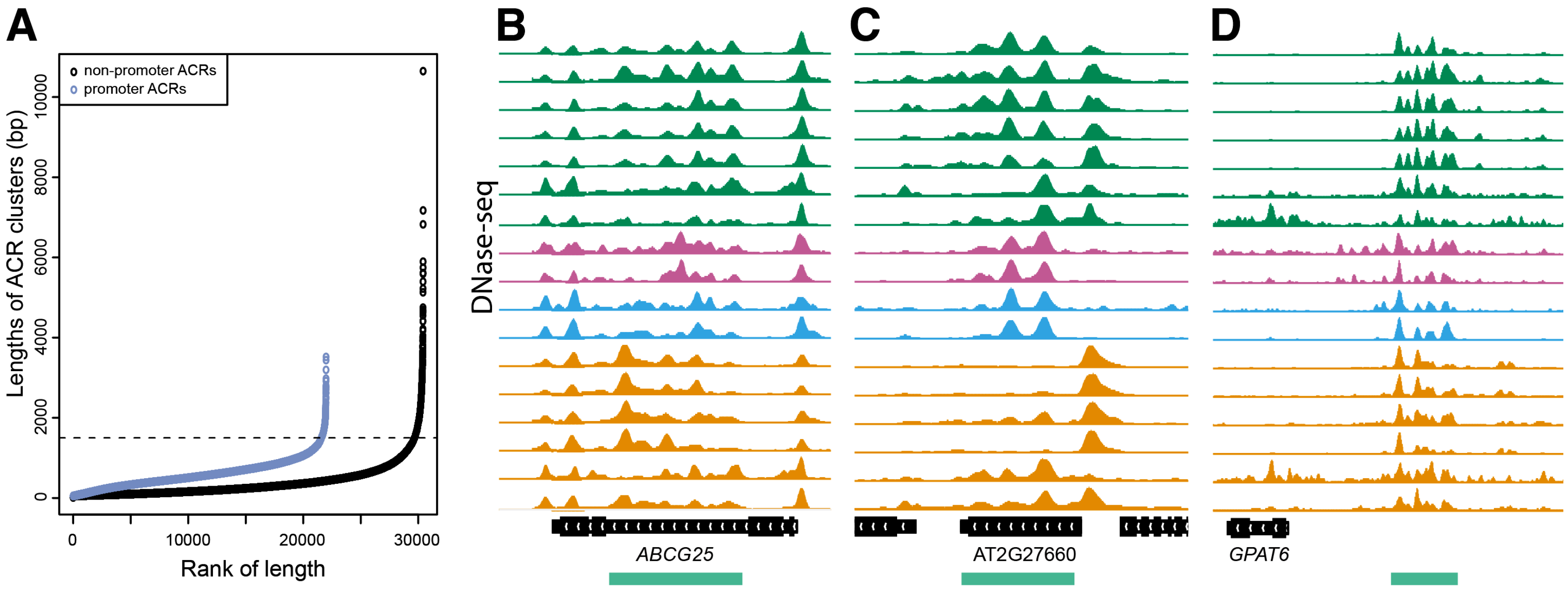

Figure S1. Lengths of ACR clusters and representative super-enhancers in *Arabidopsis*. **(A)** Distribution of the sizes of ACR clusters. **(B)** A representative super-enhancer located in an intron of gene *ABCG25*. **(C)** A representative super-enhancer located in the single exon of gene AT2G27660. **(D)** A super-enhancer is located 4.8 kb away from the TSS of gene *GPAT6*. The colors and order of the DNase-seq samples in (B-D) are the same as Figure 1A.

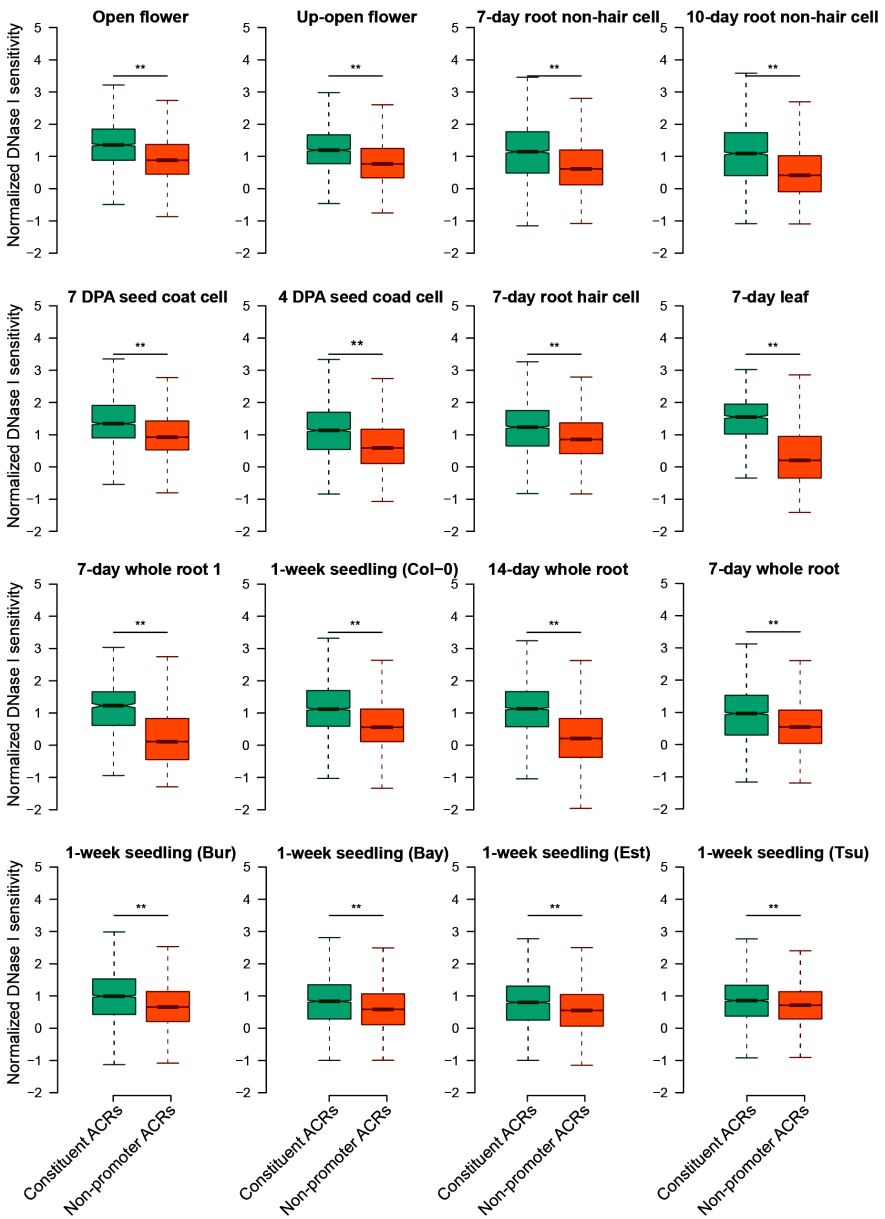

Figure S2. DNase I sensitivity associated with the constituent ACRs of SEs and other non-promoter ACRs across 16 DNase-seq samples. The DNase I sensitivity were normalized and plotted as boxplot. The significance between constituent ACRs and other non-promoter ACRs was determined by Mann-Whitney test. "**" indicates *P*-value less than 0.01.

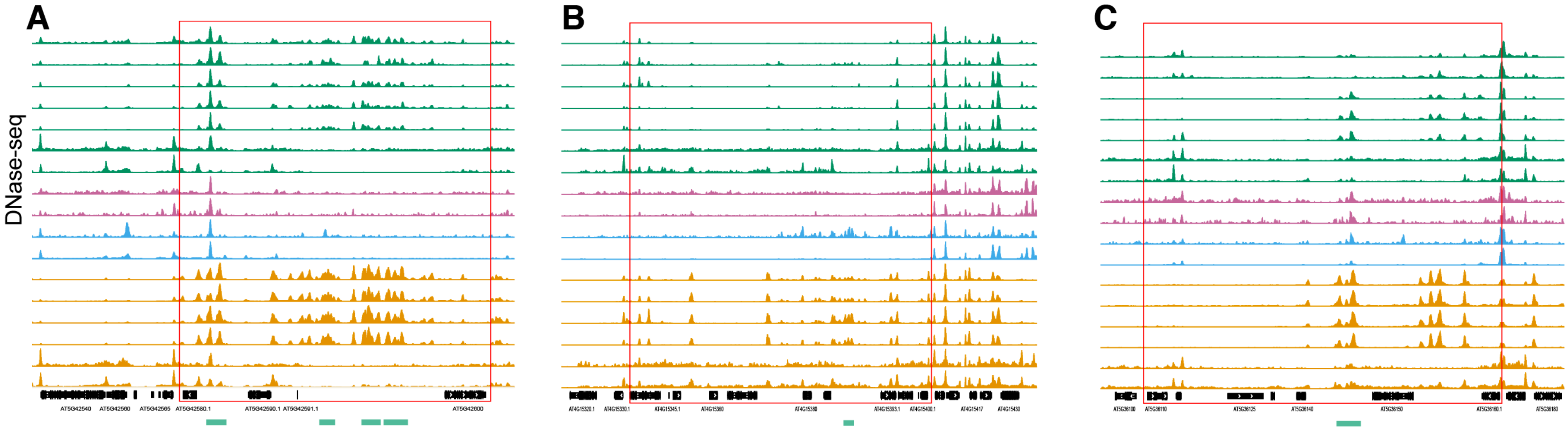

Figure S3. Super-enhancers located within metabolic gene clusters. **(A)** Four super-enhancers identified within the Marneral gene cluster. **(B)** A super-enhancer located within the Arabidiol gene cluster. **(C)** A super-enhancer located within Tirucalladienol gene cluster. Red rectangles mark the gene clusters. Green bars mark the genomic positions of the super-enhancers. The colors and order of the DNase-seq samples are the same as Figure 1A.

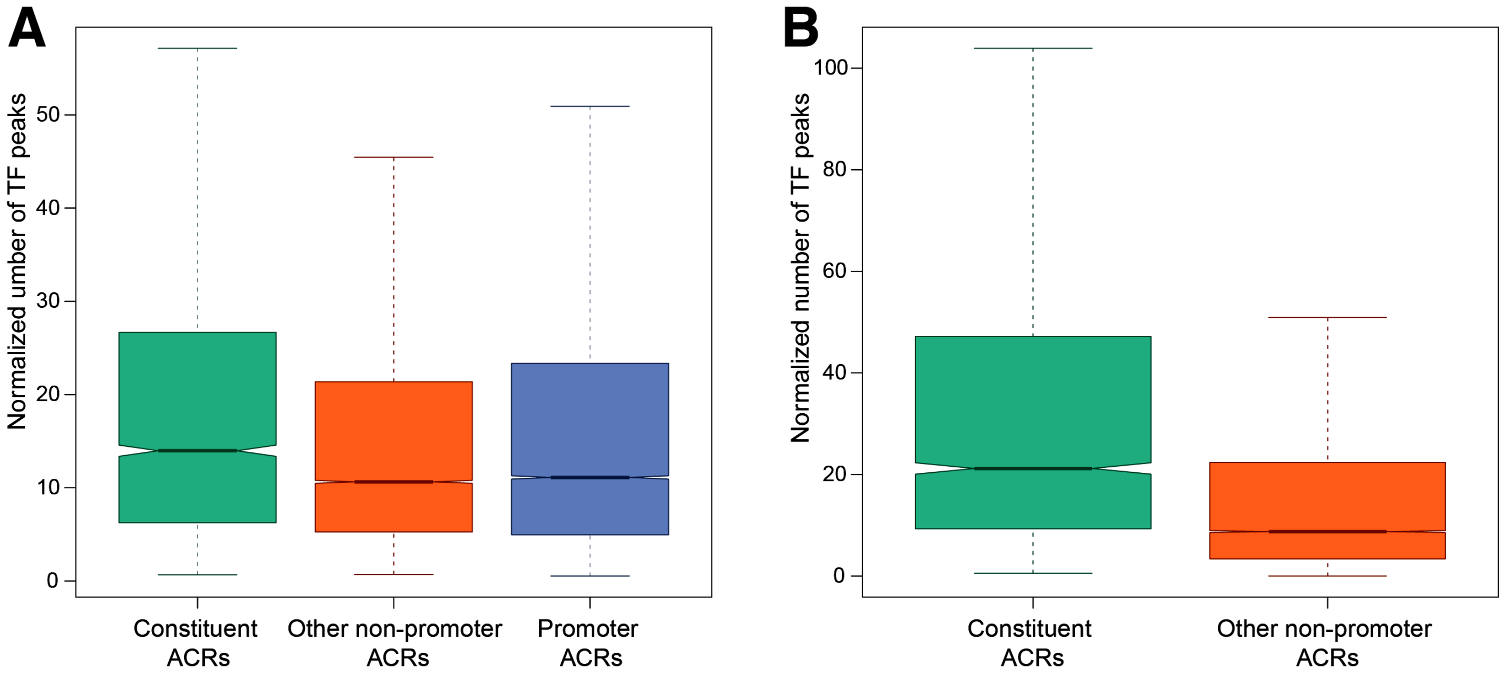

Figure S4. Number of TFs associated with the three classes of ACRs normalized by the size and distance of the ACRs to their closest genes. **(A)** The Number of TFs associated with the three classes of ACRs normalized by ACR sizes. **(B)** The Number of TFs associated with Constituent ACRs and other non-promoter ACRs normalized by their distances to the closest genes. The promoter ACRs were not included in the analysis due to their extreme close distances to genes.

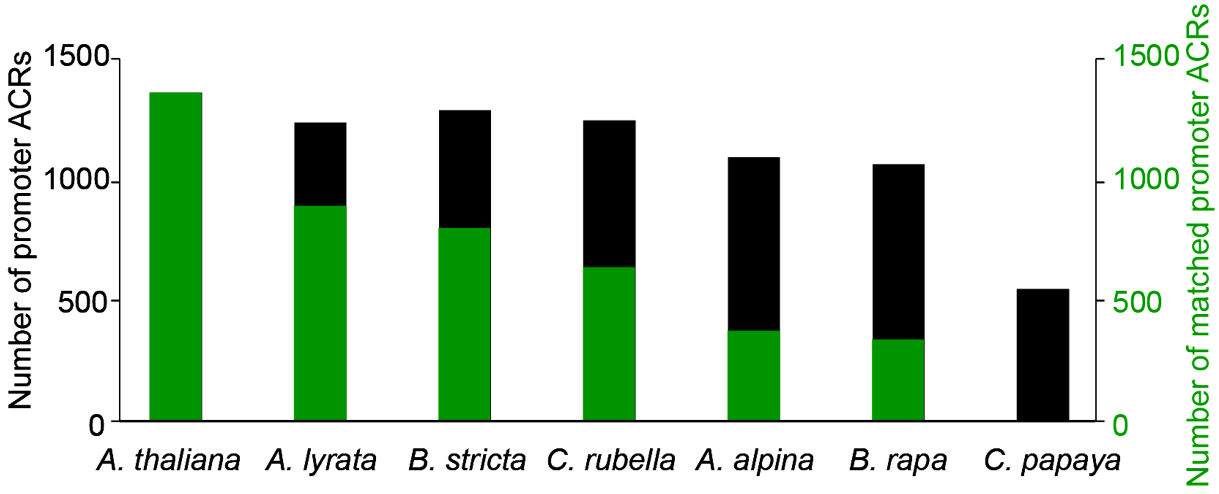

Figure S5. Sequence conservation of promoter ACRs among six Brassicaceae species. The sequences of promoter ACRs associated with syntenic gene pairs in Figure 6A were aligned to the orthologous genomic position (1 kb upstream of genes) in each of the six target species. Numbers of promoter ACRs and matched promoter ACRs in each species were calculated and represented as bar plot.

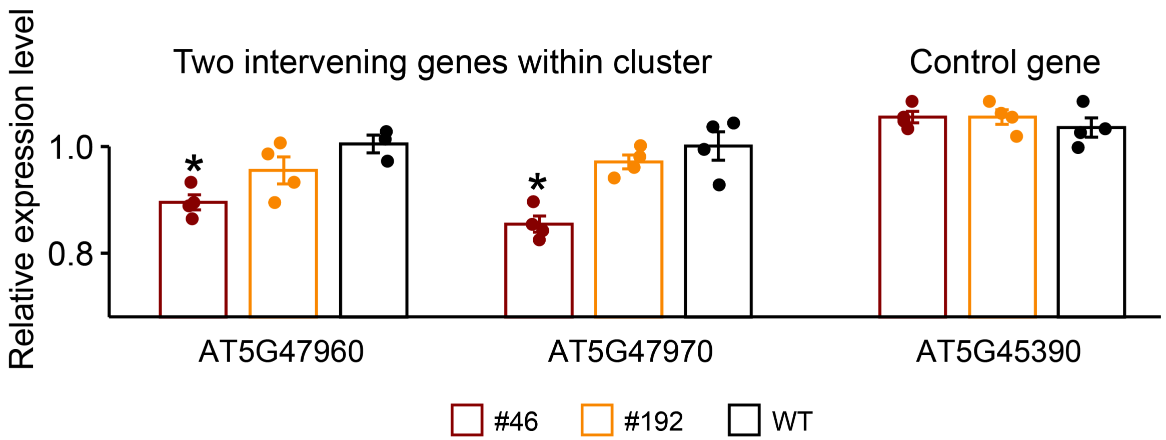

Figure S6. Relative expression levels of three non-thalianol genes in deletion lines #46 and #192 at the 4-day seedling stage. AT5G47960 and AT5G47970 are two intervening genes within the thalianol gene cluster. AT5G45390 is a gene located outside of the TAD spanning the thalianol gene cluster.

Table S1. DNase-seq datasets used in super enhancer identification and analysis.

Tissue	Stage	SRA run	Reference
leaf	14day	SRR388659,SRR388658,SRR388657	Genome-wide identification of regulatory DNA elements and protein-binding footprints using signatures of open chromatin in Arabidopsis, Plant Cell, 2012 Jul;24(7):2719-31
leaf	1week	SRR2297459	DNase I SIM: A Simplified In-Nucleus Method for DNase I Hypersensitive Site Sequencing, Methods Mol Biol. 2017;1629:141-154
seedling (col-0)	1week	SRR1049778	Mapping and dynamics of regulatory DNA and transcription factor networks in A. thaliana, Cell Rep, 2014 Sep 25;8(6):2015-2030
seedling (tsu-1)	1week	SRR1049803,SRR1049802	Complex Relationships between Chromatin Accessibility, Sequence Divergence, and Gene Expression in Arabidopsis thaliana, Mol Biol Evol. 2018 Apr; 35(4): 837–854.
seedling (Est-1)	1week	SRR1049800,SRR1049801	Complex Relationships between Chromatin Accessibility, Sequence Divergence, and Gene Expression in Arabidopsis thaliana, Mol Biol Evol. 2018 Apr; 35(4): 837–854.
seedling (Bay-0)	1week	SRR1049799,SRR1049798	Complex Relationships between Chromatin Accessibility, Sequence Divergence, and Gene Expression in Arabidopsis thaliana, Mol Biol Evol. 2018 Apr; 35(4): 837–854.
seedling (Bur-0)	1week	SRR1049797,SRR1049796	Complex Relationships between Chromatin Accessibility, Sequence Divergence, and Gene Expression in Arabidopsis thaliana, Mol Biol Evol. 2018 Apr; 35(4): 837–854.
up-open flowers	-	SRR1049813	Mapping and Dynamics of Regulatory DNA in Maturing Arabidopsis thaliana Siliques.", Front Plant Sci, 2019;10:1434
open flowers	-	SRR1049812	Mapping and Dynamics of Regulatory DNA in Maturing Arabidopsis thaliana Siliques.", Front Plant Sci, 2019;10:1434
seed coat cell	4 days past anthesis	SRR2103595	Mapping and Dynamics of Regulatory DNA in Maturing Arabidopsis thaliana Siliques, Front Plant Sci, 2019;10:1434
seed coat cell	7 days past anthesis	SRR2103593	Mapping and Dynamics of Regulatory DNA in Maturing Arabidopsis thaliana Siliques, Front Plant Sci, 2019;10:1434
whole root	1 week	SRR2297460	DNase I SIM: A Simplified In-Nucleus Method for DNase I Hypersensitive Site Sequencing, Methods Mol Biol. 2017;1629:141-154
root non-hair cell	7 day	SRR2101856	Mapping and dynamics of regulatory DNA and transcription factor networks in A. thaliana, Cell Rep, 2014 Sep 25;8(6):2015-2030

root non-hair cell	10 day	SRR2101858	Mapping and dynamics of regulatory DNA and transcription factor networks in A. thaliana, Cell Rep, 2014 Sep 25;8(6):2015-2030
whole root	7 day	SRR2101859	Mapping and dynamics of regulatory DNA and transcription factor networks in A. thaliana, Cell Rep, 2014 Sep 25;8(6):2015-2030
root hair cell	7 day	SRR2103597	Mapping and dynamics of regulatory DNA and transcription factor networks in A. thaliana, Cell Rep, 2014 Sep 25;8(6):2015-2030
whole root	2 week	SRR10051094	Local Changes in Chromatin Accessibility and Transcriptional Networks Underlying the Nitrate Response in Arabidopsis Roots, Mol Plant. 2019 Dec 2;12(12):1545-1560

Table S2. Comparison of the expression levels between SE-cognate genes and genes associated with other non-promoter ACRs in 79 tissues.

Tissue name	*P* -value*
Seedling hypocotyl	3.56E-09
Seedling meristem	0.001987579
Seedling cotyledons	4.21E-05
Seedling root	2.44E-12
Root apex	4.37E-13
Root	1.66E-17
SAM_7day	0.006056855
SAM_8day	0.004381157
SAM_9day	0.007542924
SAM_10day	0.055504723
SAM_11day	0.0451741
SAM_128day	0.010074266
SAM_13day	0.040599616
SAM_14day	0.037122821
SAM_15day	0.071736812
SAM_16day	0.051579076
Petiole of the young leaf	0.029432753
Leaf blade of the young leaf	0.021757743
Petiole, intermediate 1	0.006098841
Leaf blade, intermediate 1	0.003813659
Petiole, intermediate 2	5.32E-05
Leaf vein, intermediate 2	0.000247322
Leaf blade, intermediate 2	0.000230437
Petiole of the mature leaf	1.36E-10
Vein of the mature leaf	4.14E-10
Leaf blade of the mature leaf	1.62E-07
mature leaf	6.82E-09
Petiole of the senescent leaf	3.34E-14
Vein of the senescent leaf	4.97E-12
Opened anthers	1.05E-08
Carpels of the mature flower (before pollination	0.000829237
Anthers of the mature flower (before opening).	7.33E-05
Stamen filaments of the mature flower	4.05E-06
Petals of the mature flower	3.22E-07
Sepals of the mature flower	3.36E-08
Carpels of the young flower	0.010097888
Anthers of the young flower	0.000224296
Sepals of the young flower	7.01E-05
Flower 1	7.77E-08
Flower 2	1.09E-06
Flower 3	5.65E-05
Flower 4	0.000207906
Flower 5	0.000362559

Flower 6	0.000102289
Flower 7	0.000629995
Flower 8	0.000208767
Flower 9	0.001114923
Flower 10	0.004075861
Pedicel	0.001232406
Axis of the inflorescence	0.032705406
Internode	1.06E-08
seeds 1	0.010179827
seeds 3	6.57E-09
seeds 5	5.42E-08
seeds 7	0.000148077
pod of the silique 1	1.76E-09
pod of the silique 3	8.08E-10
pod of the silique 5	0.001239452
pod of the silique 7	5.94E-10
silique 2	2.33E-09
silique 4	9.41E-05
silique 6	4.53E-09
silique 8	2.84E-07
young seeds 1	0.004501001
young seeds 2	0.003829494
young seeds 3	0.004889917
young seeds 4	0.001738123
young seeds 5	0.007224454
Ovules from 6th and 7th flowers	0.01611568
Stigmatic tissue	0.010250958
Carpel of 6th and 7th flowers	0.001345312
dry seeds	0.780039556
germinating seeds 1	0.238559744
germinating seeds 2	4.09E-05
germinating seeds 3	5.33E-12
Seeds of first yellowing silique	0.295088049
pod of the senescent silique 1	9.22E-09
senescent silique 2	0.0004122
senescent internode	9.56E-12

*** Mann-Whitney test. Gray shade marks the tissues in which the exprssion levels are the same between the two group of genes.**

Table S3. GO enrichment of super enhancers.

GO enrichment of super enhancers comparing to promoter ACRs

GO term	Description	q-value	Catelog
GO:0065009	regulation of molecular function	2.47E-17	biological_process
GO:0030154	multicellular organism development	3.11E-02	biological_process
GO:0003700	DNA-binding transcription factor activity	1.06E-17	molecular_function
GO:0005515	protein binding	1.10E-03	molecular_function

GO enrichment of super enhancers comparing to other non-promoter ACRs

GO term	Description	q-value	Catelog
GO:0065009	regulation of molecular function	6.85E-11	biological_process
GO:0030154	multicellular organism development	2.31E-02	biological_process
GO:0003700	DNA-binding transcription factor activity	3.57E-11	molecular_function
GO:0005515	protein binding	4.00E-02	molecular_function

Table S4. List of transcription factors that are associated with root development and contain binding motifs within the SE related to thalianol gene cluster.

Gene ID	Gene name	Reference
AT1G69780	ATHB13	A predictive coexpression network identifies novel genes controlling the seed-to-seedling phase transition in Arabidopsis thaliana,Plant Physiol. 2016 Apr; 170(4): 2218–2231.
AT2G40950	BZIP17	Salt stress responses in Arabidopsis utilize a signal transduction pathway related to endoplasmic reticulum stress signaling, Plant J. 2007 Sep;51(5):897-909
AT5G47370	HAT2	The HAT2 gene, a member of the HD-Zip gene family, isolated as an auxin inducible gene by DNA microarray screening, affects auxin response in Arabidopsis, Plant J. 2002 Dec;32(6):1011-22
AT5G18560	PUCHI	The Auxin-Regulated AP2/EREBP Gene PUCHI Is Required for Morphogenesis in the Early Lateral Root Primordium of Arabidopsis, Plant Cell. 2007 Jul;19(7):2156-68
AT4G04450	WRKY23	The lncRNA APOLO interacts with the transcription factor WRKY42 to trigger root hair cell expansion in response to cold, Mol Plant. 2021 Jun 7;14(6):937-948
AT4G00730	ANL2	ANTHOCYANINLESS2, a homeobox gene affecting anthocyanin distribution and root development in Arabidopsis, Plant Cell. 1999 Jul;11(7):1217-26
AT4G30080	ARF16	Control of Root Cap Formation by MicroRNA-Targeted Auxin Response Factors in Arabidopsis, Plant Cell. 2005 Aug;17(8):2204-16
AT5G51190	ERF105	Characterisation of the ERF102 to ERF105 genes of Arabidopsis thaliana and their role in the response to cold stress, Plant Mol Biol. 2020 Jun;103(3):303-320
AT5G07310	ERF115	ETHYLENE RESPONSE FACTOR 115 integrates jasmonate and cytokinin signaling machineries to repress adventitious rooting in Arabidopsis, New Phytol. 2020 Dec;228(5):1611-1626
AT5G47220	ERF2	Repressor- and Activator-Type Ethylene Response Factors Functioning in Jasmonate Signaling and Disease Resistance Identified via a Genome-Wide Screen of Arabidopsis Transcription Factor Gene Expression, Plant Physiol. 2005 Oct;139(2):949-59
AT1G73360	DHG11/EDT1	Activated Expression of an Arabidopsis HD-START Protein Confers Drought Tolerance with Improved Root System and Reduced Stomatal Density, Plant Cell. 2008 Apr;20(4):1134-51; Characterization of the Class IV Homeodomain-Leucine Zipper (HD-ZIP IV) Gene Family in Arabidopsis, Plant Physiol. 2006 Aug; 141(4): 1363–1375.
AT5G03150	JKD	Arabidopsis JACKDAW and MAGPIE zinc finger proteins delimit asymmetric cell division and stabilize tissue boundaries by restricting SHORT-ROOT action, Genes Dev. 2007 Sep 1; 21(17): 2196–2204.
AT1G03840	MGP/IDD3	Arabidopsis JACKDAW and MAGPIE zinc finger proteins delimit asymmetric cell division and stabilize tissue boundaries by restricting SHORT-ROOT action, Genes Dev. 2007 Sep 1; 21(17): 2196–2204.

AT1G19850	MP/IAA24/ARF5	Auxin response cell-autonomously controls ground tissue initiation in the early Arabidopsis embryo, Proc Natl Acad Sci U S A. 2017 Mar 21; 114(12): E2533–E2539.
AT5G17800	MYB56/BRAVO	TOPLESS mediates brassinosteroid control of shoot boundaries and root meristem development in Arabidopsis thaliana, Development. 2017 May 1;144(9):1619-1628
AT2G23290	MYB70	MYB70 modulates seed germination and root system development in Arabidopsis, iScience. 2021 Oct 7;24(11):103228
AT1G34670	MYB93	AtMYB93 is a novel negative regulator of lateral root development in Arabidopsis, New Phytol. 2014 Sep;203(4):1194-1207
AT3G55370	OBP3	Characterization of salicylic acid-responsive, Arabidopsis Dof domain proteins: overexpression of OBP3 leads to growth defects, Plant J. 2000 Feb;21(4):329-39
AT1G71696	SOL1	Root-Specific CLE19 Overexpression and the sol1/2 Suppressors Implicate a CLV-like Pathway in the Control of Arabidopsis Root Meristem Maintenance, Curr Biol. 2003 Aug 19;13(16):1435-41
AT1G51220	WIP5	Genetic control of distal stem cell fate within root and embryonic meristems, Science. 2015 Feb 6;347(6222):655-9
AT2G47260	WRKY23	Transcription factor WRKY23 assists auxin distribution patterns during Arabidopsis root development through local control on flavonol biosynthesis, Proc Natl Acad Sci U S A. 2012 Jan 31;109(5):1554-9

Table S5. Numbers of predicted TF-binding sites identified within the SE and in the promoter-associated ACRs of the seven thalianol genes.

TF name	SE	THAA1 promoter	THAA2 promoter	THAH promoter*	THAO promoter	THAS promoter
HAT2	14	0	0	2	0	0
ATHB20	13	1	0	2	0	2
ATHB13	12	0	0	2	0	2
ANL2	10	2	0	1	0	0
ATHB53	10	1	0	2	0	2
NUC	7	2	1	1	0	2
MGP	7	2	2	2	1	2
JKD	7	2	2	2	0	2
MYB56	6	0	0	0	0	2
EDT1	5	1	0	0	0	0
AtMYB93	4	0	0	0	0	1
BZIP28	4	1	0	0	0	0
MYB33	4	0	0	0	0	0
ATHB23	4	1	0	0	0	0
OBP3	4	2	2	2	2	2
WRKY42	4	2	2	0	2	0
MYB93	4	1	0	0	0	1
AGL16	4	2	0	0	0	0
IDD5	3	1	2	2	0	2
WIP5	2	2	0	0	0	0
BZIP17	2	0	0	0	0	0
HDG11	1	0	0	0	0	0
AT2G43140	1	0	0	0	0	1
IDD9	1	1	0	0	0	0
HRS1	1	1	0	0	0	0
MYB70	1	0	0	0	1	0
OBP2	1	1	0	1	1	0
At1g68670	1	1	0	0	0	0
WRKY33	1	2	2	0	1	1
MYB77	1	0	0	0	1	1
NAC012	1	0	0	0	0	0
ASL18	0	0	0	0	0	0
ATHB25	0	1	1	0	0	1
MYB23	0	2	2	0	0	2
bZIP28	0	1	0	0	0	0
U2AF35B	0	0	0	0	0	0
WRKY23	0	1	0	0	1	0
bHLH69	0	1	0	0	0	0
MYB59	0	2	0	0	0	0
AtGRF6	0	0	0	0	0	0
ABF2	0	2	0	0	0	0
KNAT4	0	0	0	0	0	1
RKD2	0	0	0	0	2	0
PLT3	0	0	0	0	0	0
SPL7	0	1	1	0	0	0
HHO2	0	1	0	0	0	0

ZHD1	0	1	1	0	0	1
AIL6	0	0	0	0	0	0
GBF3	0	2	0	0	0	0
SPL11	0	1	0	1	0	0
ARF16	0	2	1	0	1	2
HHO3	0	1	0	0	0	0
KNAT3	0	0	0	0	0	1
At1g25550	0	1	0	0	0	1
LRL2	0	1	0	0	0	0
At1g13300	0	1	0	0	0	1
SMB	0	0	2	0	0	1

*** Each promoter is defined as the ACR that overlaps with 500 bp upstream of the transcription start site of each gene. For THAH, there is no ACR at the promoter region. Instead, the ACR located in the first intron was used for the motif analysis.**

Table S6. List of sgRNAs used in CRISPR/Cas editing.

Primer name	Sequence
gRNA1-top	ATTGCATCAGTTGTTACGATCTCA
gRNA1-bottom	AAACTGAGATCGTAACAACTGATG
gRNA2-top	ATTGTTATTATCAAATGGAACGCT
gRNA2-bottom	AAACAGCGTTCCATTTGATAATAA
gRNA3-top	ATTGCCTATTGGGAAACCAAAAAC
gRNA3-bottom	AAACGTTTTTGGTTTCCCAATAGG

Table S7. Primers used for quantitative real-time polymerase chain reaction.

Primer name	Sequence
AT5G47950-qPCR-F	GGCCGGTAGAGTCAATGGAG
AT5G47950-qPCR-R	TTCGGTATCCGGGGATCTCA
AT5G47960-qPCR-F	CGGTGTCGAGTTTCAGACAAG
AT5G47960-qPCR-R	GTCACCGCTCGATACCGTTC
AT5G47970-qPCR-F	CAAGGCGACAGGTTCTTGAAA
AT5G47970-qPCR-R	TCCAGACTCCGAGTGGAACA
AT5G47980-qPCR-F	ACACTCGCACCAGGAGAATC
AT5G47980-qPCR-R	CAGTGAGAGACGCAGATCCC
AT5G47990-qPCR-F	TTGTCCCAATCCACCAGTCTT
AT5G47990-qPCR-R	AGGAGACGAGGACTATGGGG
AT5G48000-qPCR-F	AATGTGCGGAGACTTCCTCG
AT5G48000-qPCR-R	AGCGACGACCAAAATAGCGA
AT5G48010-qPCR-F	AATTTGTGGCTCCCCCAACA
AT5G48010-qPCR-R	CATCGGTTTCGTGTTTGCGT
AT5G45390-qPCR-F	GACGATTTCGTCGCTGATGC
AT5G45390-qPCR-R	GCACTGAGAGAACCACCAGG
AtUBC21-qPCR-F	TGCAGTTGACAATTCGTTCTC
AtUBC21-qPCR-R	CGGTCCATTTGAATATGTTGGT
